# Pyrophosphate‐Containing Calcium Phosphates Negatively Impact Heterotopic Bone Quality

**DOI:** 10.1002/adhm.202405171

**Published:** 2025-05-22

**Authors:** Martina Jolic, Isabella Åberg, Omar Omar, Håkan Engqvist, Thomas Engstrand, Anders Palmquist, Peter Thomsen, Furqan A. Shah

**Affiliations:** ^1^ Department of Biomaterials Institute of Clinical Sciences, Sahlgrenska Academy University of Gothenburg Box 412 Gothenburg 405 30 Sweden; ^2^ Department of Biomedical Dental Sciences College of Dentistry Imam Abdulrahman Bin Faisal University P.O. Box 1982 Dammam 314 41 Saudi Arabia; ^3^ Department of Materials Science and Engineering Uppsala University Box 35 Uppsala 751 03 Sweden; ^4^ Department of Molecular Medicine and Surgery Karolinska University Hospital Stockholm 171 76 Sweden

**Keywords:** calcium phosphate, calcium pyrophosphate, heterotopic bone, heterotopic ossification, in vivo, osteoinduction

## Abstract

In bone, critical size defects pose substantial challenge in maxillofacial and orthopedic reconstructions as they are incapable of spontaneous regeneration. In such cases, autografts, allografts, and bone graft substitutes are used. Calcium phosphates (CaP) are widely used bone graft substitutes due to their biocompatibility, osteoconductive properties, and potential for osteoinductivity. CaP materials containing monetite, beta‐tricalcium phosphate (β‐TCP), and a small amount of calcium pyrophosphate (Ca‐PP) possess both osteoconductive and osteoinductive properties. However, the role of Ca‐PP in osteoinduction and material degradation remains unexplored. This study investigates heterotopic bone formation in response to five CaP compositions, maintaining a constant monetite to β‐TCP ratio, with varying amounts of Ca‐PP (0–12.5%). Twelve adult female sheep (*Ovis aries*) are subcutaneously implanted with constructs made of six CaP tiles interconnected by a Ti6Al4V frame and a control implant. Histological analysis, backscattered electron scanning electron microscopy, and Raman spectroscopy of samples retrieved at 12‐ and 52 weeks reveal that Ca‐PP does not hinder heterotopic bone formation and minimally impacts CaP degradation. While monetite and β‐TCP transform into apatite, the Ca‐PP phase remains unchanged. The addition of Ca‐PP to the CaP influences heterotopic bone quality and inflammatory response during tissue regeneration.

## Graphical abstract

The role of calcium pyrophosphate (Ca‐PP) in heterotopic bone formation and material degradation is investigated using calcium phosphate (CaP) compositions with varying Ca‐PP content. Results show that Ca‐PP does not impede heterotopic bone formation, minimally affects CaP degradation at non‐osseous sites, and increases phagocytosis. Higher Ca‐PP concentrations are associated with lower *de novo* bone quality.

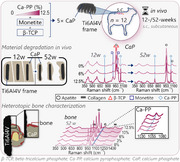

## 1 Introduction

Bone possesses a remarkable capacity for self‐repair and regeneration. However, certain conditions such as injury, tumor resection, or debridement of infected tissue can result in critical‐size defects incapable of spontaneous regeneration.^[^ ^1^ ^]^ In such cases, reconstruction is undertaken using natural bone grafts (autologous and allogenic) or synthetic bone graft substitutes (e.g., calcium sulfates, calcium phosphates, bioactive glasses).^[^ ^2^ ^]^ Currently, autologous bone grafts are considered the “gold standard” in bone repair and reconstruction, meeting both the mechanical and biological requisites of an ideal replacement material. However, their limited availability, high resorption, high infection rates, and associated morbidities highlight the importance of developing synthetic bone graft substitutes.^[^ ^3^ ^]^ Among bone graft substitutes, calcium phosphate (CaP) materials are extensively used due to their similarity to bone mineral, biocompatibility, biodegradability, osteoconductive, and potential osteoinductive properties.^[^ ^4^ ^]^

The concept of osteoinduction was first described by Urist in 1965, who demonstrated bone formation upon implantation of demineralized bone matrix in the muscles of mice, rats, rabbits, and guinea pigs.^[^ ^5^ ^]^ Consequently, an osteoinductive material was defined as a material supporting the osteogenic differentiation of progenitor cells.^[^ ^6^ ^]^ Currently, intrinsically osteoinductive materials are defined by their ability to mineralize in vivo, the presence of pores that can accommodate vascularization, and their ability to create a local environment depleted of Ca^2+^ and PO_4_^3^⁻.^[^ ^7^ ^]^ For a material to be considered osteoinductive, it should be able to induce bone formation heterotopically.^[^ ^8^ ^]^ While all osteoinductive materials are osteoconductive, the reverse is not always true. Rather, osteoconductive materials serve as scaffolds for new bone growth, with their effectiveness depending on the chemical composition and presence of macro‐ and microporosities.^[^ ^9^ ^]^ This distinction is especially relevant when working with CaP materials, as their bone regeneration capacity relies heavily on their osteoconductive as well as osteoinductive properties. Assessing the osteoinductive capacity of materials is essential for evaluating their efficacy, biocompatibility, integration with bone tissue, ability to enhance healing, and clinical success.

Various CaP compositions are already in clinical use, resulting in promising outcomes and even outperforming autologous bone grafts in certain cases.^[^ ^10^ ^]^ Ongoing research into CaP materials aims to harness both their osteoconductive and osteoinductive properties to optimize bone regeneration strategies. Beta‐tricalcium phosphate (β‐TCP), recognized for both osteoconductive and osteoinductive properties, is widely used as a bone graft substitute in dental, orthopedic, and drug delivery applications.^[^ ^11^ ^]^ Biphasic calcium phosphate (BCP) bioceramics, consisting of varying proportions of β‐TCP and hydroxy(l)apatite (HAp), are among the most studied CaP‐based bone graft substitutes, although the optimal β‐TCP‐to‐HAp ratio remains debated. Higher β‐TCP levels associated with faster biodegradation and higher levels of HAp impart better mechanical properties.^[^ ^12^ ^]^ The osteoinductive potential of CaP materials, particularly BCPs having a β‐TCP‐to‐HAp ratio of 40/60, has been demonstrated in sheep models, with intramuscular bone formation observed after six months in vivo.^[^ ^13^ ^]^ Additionally, monetite has shown favorable osteoconductive properties in various animal models as a component of moldable cements, coatings, granules, and scaffolds.^[^ ^14^ ^]^ Another CaP phase of interest, calcium pyrophosphate (Ca‐PP), has been shown to stimulate bone formation as a component of bone cements in the treatment of bone defect models.^[^ ^15^ ^]^ Porous Ca‐PP scaffolds have displayed comparable osteoconductivity and improved in vivo degradation relative to HAp scaffolds in canine and rabbit models.^[^ ^16^ ^]^ This is somewhat surprising, considering that ions of pyrophosphate are known inhibitors of mineralization and mineral crystal growth, and the formation of Ca‐PP crystals in articular cartilage is associated with inflammation, chondrocyte catabolism, and articular damage.^[^ ^17^ ^]^ Moreover, the biological performance of CaP‐based materials can be easily modified through the inclusion of different phases, ion doping, adjustment of pore sizes, or incorporation of growth factors.^[^ ^4^, ^18^ ^]^

We have previously demonstrated that a monetite‐based CaP material containing β‐TCP and Ca‐PP promotes bone regeneration in large cranial defects and guides bone formation beyond the skeletal margins.^[^ ^19^ ^]^ Furthermore, we established the osteoinductive properties of the material with similar composition at 12 weeks in vivo. Here, building on these findings, we investigated a series of monetite‐based CaP compositions with varying amounts of added Ca‐PP over 12‐ and 52‐week periods in vivo, to evaluate the short‐ and long‐term effects of Ca‐PP on osteoinductive capacity and degradation of CaP materials. Through detailed histological analysis and extensive Raman spectroscopy, we characterized the effects of Ca‐PP on heterotopic bone formation and CaP material degradation.

## 2 Results

### 2.1 Histology and Histomorphometry

**Figure 1:**
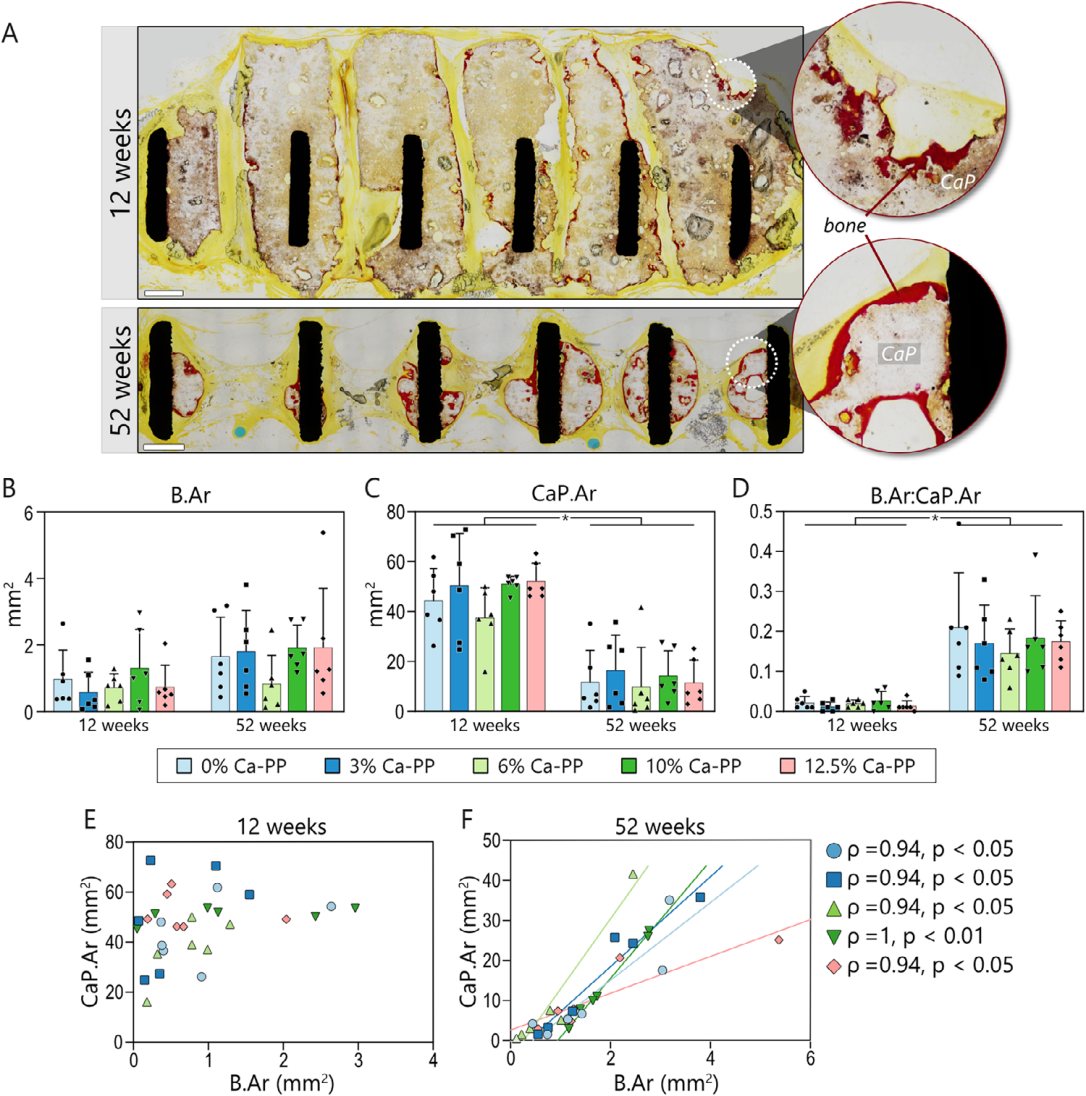
Heterotopic bone and CaP area in vivo. A) Overviews of representative undecalcified histological sections stained with Van Gieson's stain at 12‐ and 52 weeks (0% Ca‐PP). Bone is stained intensely red, making it easily distinguishable from non‐mineralized soft tissue and CaP. Scale bar = 1 mm. Histomorphometry: B) heterotopic bone area (B.Ar), C) CaP area (CaP.Ar), and D) B.Ar normalized to CaP.Ar. Statistically significant difference marked with an asterisk, *p* = 0.002. E) Relationship between CaP.Ar and B.Ar measured in respective CaP compositions at 12 weeks. F) Relationship between CaP.Ar and B.Ar measured in respective CaP compositions at 52 weeks. Spearman's correlation analysis.

Undecalcified, Van Gieson‐stained histological sections were used to detect the presence of heterotopic bone and measure the areas occupied by the heterotopic bone (B.Ar) and the calcium phosphate constructs (CaP.Ar) after 12‐ and 52 weeks in vivo (Tables S2 and S3, Supporting Information). Heterotopic bone was consistently found at both time points, irrespective of the amount of Ca‐PP in the material (**Figure** **1**A,B). In contrast, no heterotopic bone formed in response to the Ti6Al4V ELI control (Figures S12, S13, Supporting Information). The measured B.Ar was comparable across all CaP material compositions at both time points, averaging 0.86 ± 0.77 mm^2^ at 12 weeks and 1.63 ± 1.19 mm^2^ at 52 weeks (Figure 1B). Although there was a trend toward higher B.Ar for all compositions at 52 weeks in vivo (*p* = 0.002), this difference was not statistically significant when comparing the two‐time points for individual CaP compositions. Nevertheless, substantial physical degradation of the CaP material occurred in vivo (Figure 1C). On average, the measured CaP.Ar was 47.09 ± 13.08 mm^2^ at 12 weeks and was significantly reduced (*p* < 0.0001) to 12.75 ± 11.93 mm^2^ by 52 weeks.

Normalizing B.Ar to CaP.Ar highlighted the time‐dependent increase in heterotopic B.Ar (Figure 1D). At both 12‐ and 52 weeks, the measured CaP.Ar was comparable between the CaP material compositions, irrespective of the Ca‐PP content. While a positive relationship was not evident between B.Ar and CaP.Ar at 12 weeks in vivo (Figure 1E), at 52 weeks, the presence and extent of heterotopic B.Ar correlated with the remaining CaP material. Spearman's correlation analysis confirmed this finding for all compositions (Figure 1F). Given that the amount of heterotopic bone formation was not influenced by the amount of Ca‐PP in the CaP material, data from all groups were combined for further analyses. Pooled data revealed a low correlation between B.Ar and CaP.Ar at 12 weeks (ρ = 0.41, *p* < 0.05) increased to a very high correlation at 52 weeks (ρ = 0.92, *p* < 0.0001).

### 2.2 Fate of CaP at a Distance from the Bone‐Material Interface

**Figure 2:**
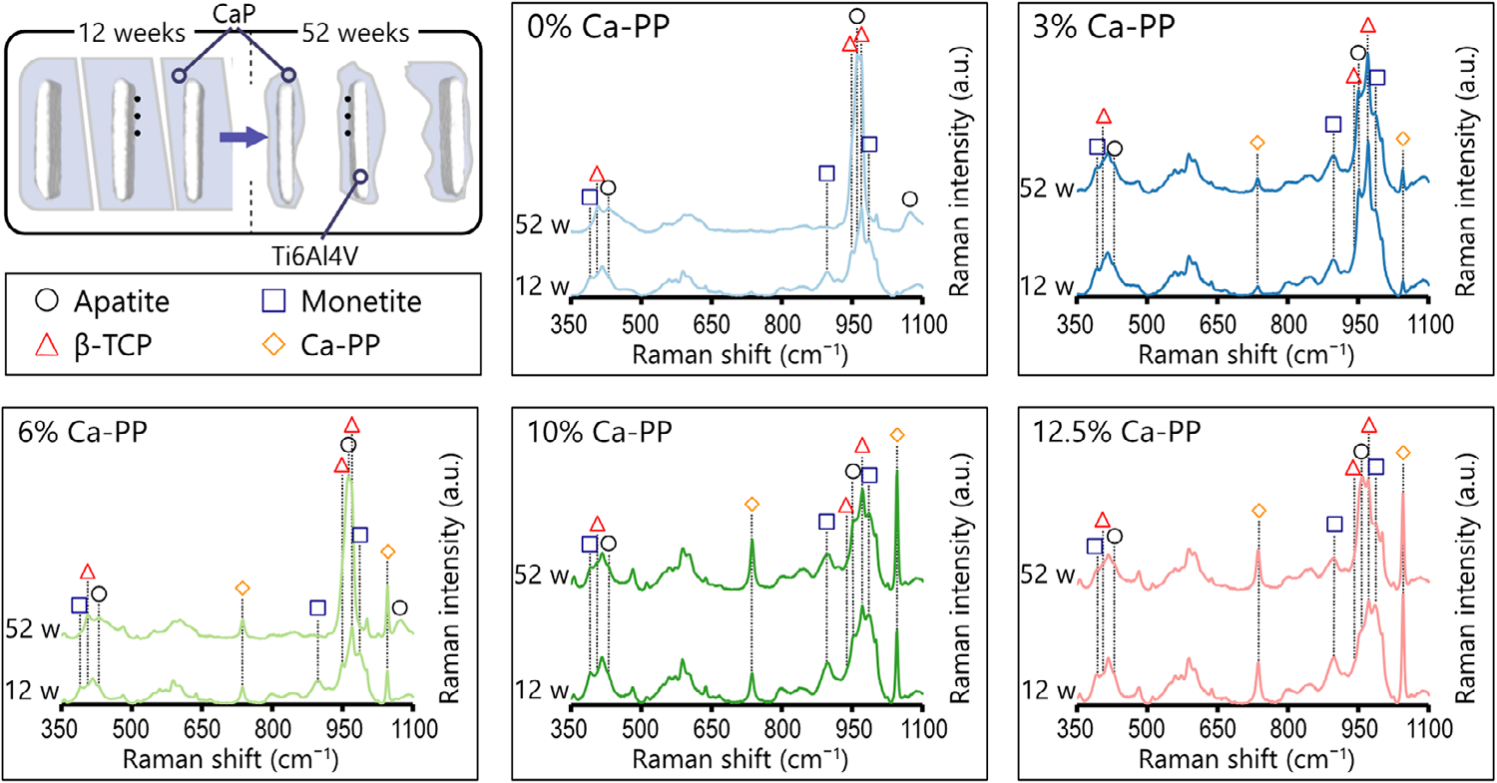
Fate of CaP in vivo. Top left: Schematic representation of significant CaP degradation in vivo. Black dots are representative of Raman spectroscopy point measurements in areas of the CaP material located furthest from the bone‐material interface. Averaged Raman spectra (*n* = 6) of CaP at 12‐ and 52 weeks. Apatite (≈431 and 960 cm⁻^1^), carbonate (≈1070 cm⁻^1^), monetite (≈391, 900, and 986 cm⁻^1^), β‐TCP (≈407, 947, and 970 cm⁻^1^), and Ca‐PP (≈732 and 1043 cm⁻^1^).

Significant physical degradation of CaP from 12‐ to 52 weeks in vivo appears to be independent of the amount of Ca‐PP originally present in the CaP constructs. To determine the expected chemical transformation of the CaP material, point measurements were made in areas of the material located furthest from the bone‐material interface using Raman spectroscopy (**Figure** **2**). After 12 weeks in vivo, distinctive spectral features representing the three constituent phases, i.e., monetite (900 and 986 cm⁻^1^ peaks), β‐TCP (947 and 970 cm⁻^1^ peaks), and Ca‐PP (732 and 1043 cm⁻^1^ peaks), were identified. The intensities of Ca‐PP peaks in the Raman spectra reflected the amount of Ca‐PP present initially (0–12.5%) in the CaP material compositions. At 12 weeks, a partial transformation of the material to apatite was evident from the peak at ≈960 cm⁻^1^ (ν_1_PO_4_^3−^) in the Raman spectra. The spectra of the respective compositions were largely comparable between the two‐time points (Figure 2). However, at 52 weeks in vivo, the 0% and 6% Ca‐PP compositions showed more pronounced chemical transformation of CaP. In these, carbonated apatite (CHAp) was formed via the depletion of the monetite phase, as interpreted from the absence of peaks at 900 and 986 cm⁻^1^ and the emergence of the peak ≈1070 cm⁻^1^ (ν_1_CO_3_^2−^). Conversely, the β‐TCP and Ca‐PP phases were still detectable within the averaged spectra (Figure 2).

### 2.3 Fate of the CaP at the Bone‐Material Interface

**Figure 3:**
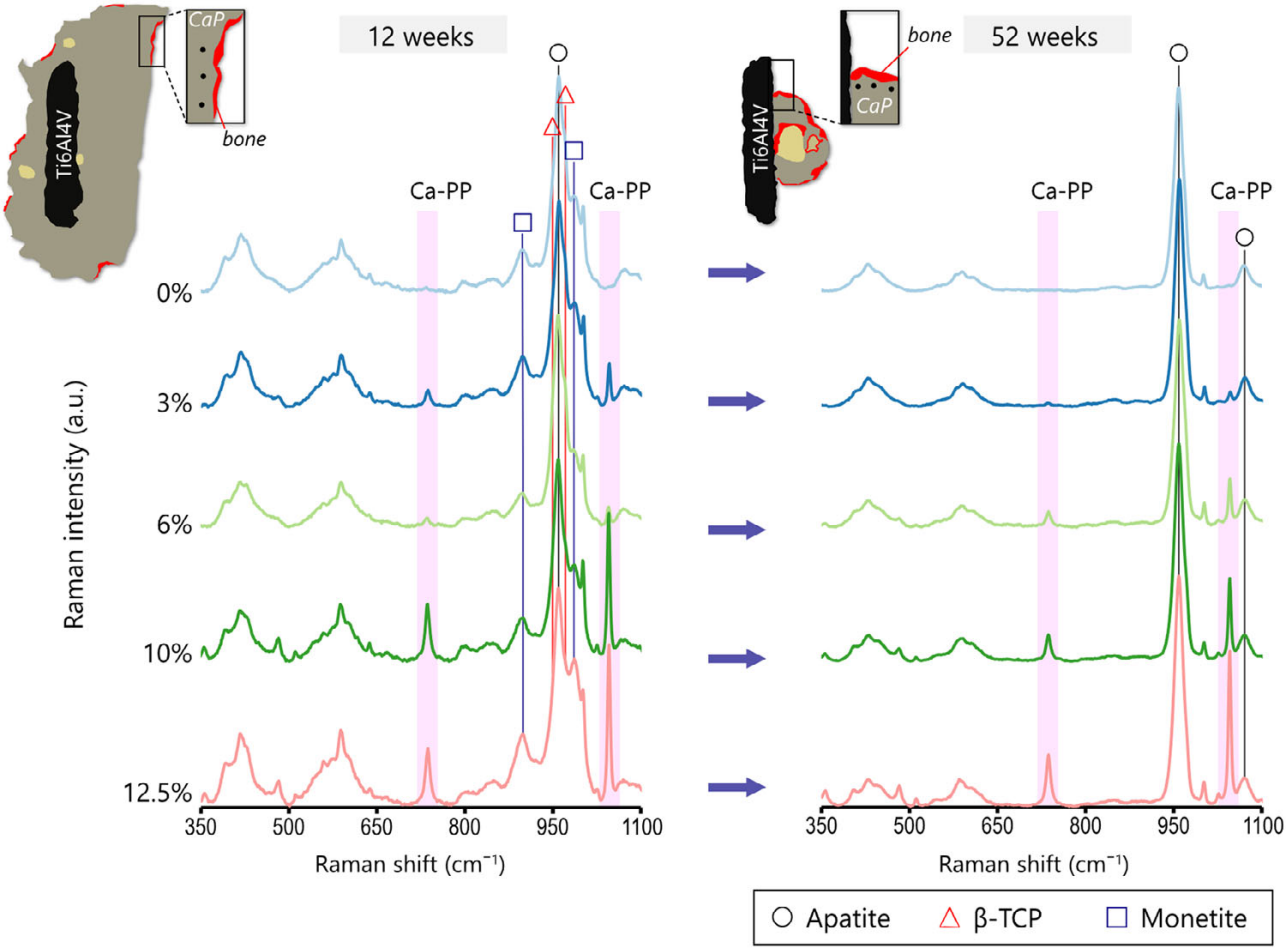
Chemical transformation of CaP material components at the interface with heterotopic bone. Top: Schematic representation of CaP constructs at 12‐ and 52 weeks in vivo and the location of Raman spectroscopy point measurements (black dots) made in areas of the CaP material in close proximity (50–100 µm) to the bone interface. Averaged Raman spectra (*n* = 6) of CaP at 12‐ and 52 weeks. Apatite (≈960 cm⁻^1^), carbonate (≈1070 cm⁻^1^), monetite (≈900 and 986 cm⁻^1^), β‐TCP (≈947 and 970 cm⁻^1^), and Ca‐PP (≈732 and 1043 cm⁻^1^).

**Figure 4:**
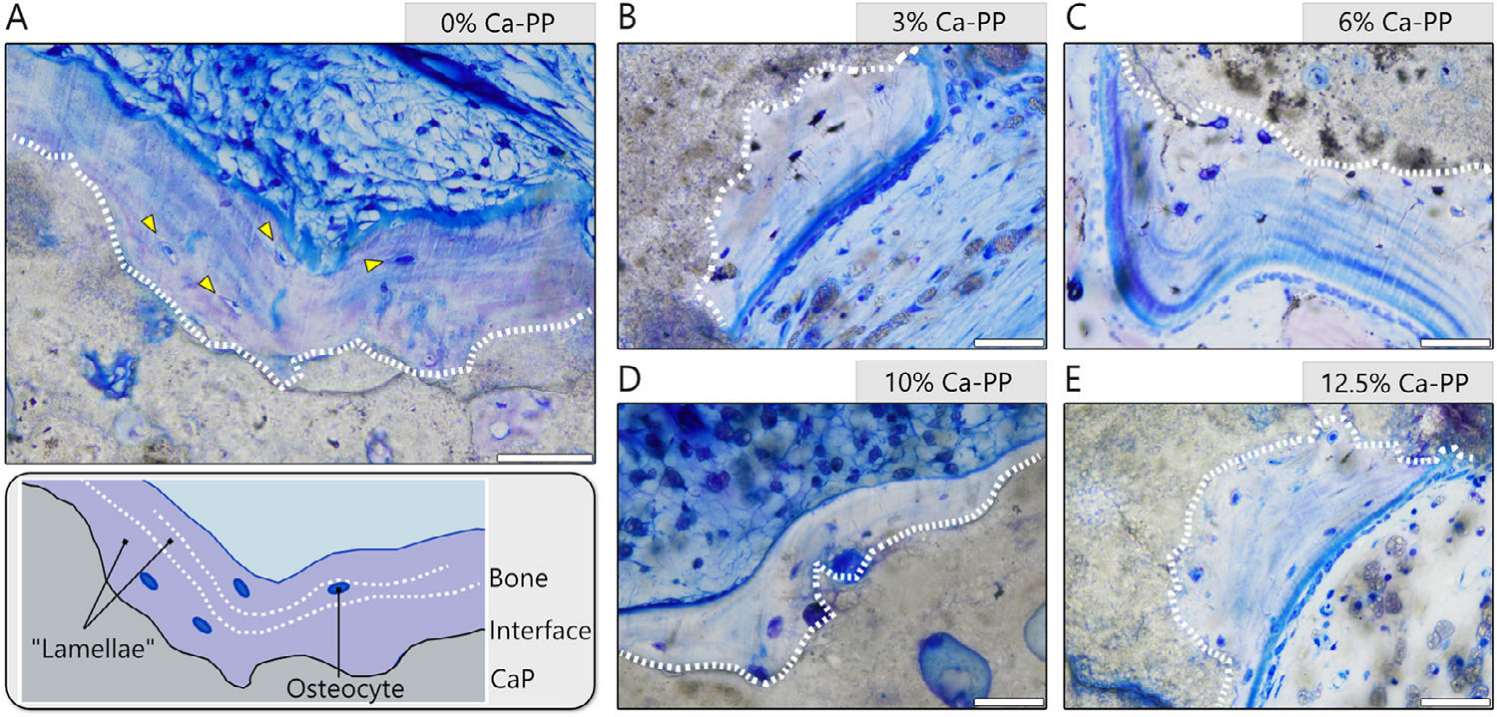
The lamellar‐like appearance of heterotopic bone at 12 weeks in vivo. Representative, undecalcified, toluidine blue stained histological sections for: A) 0% Ca‐PP, B) 3% Ca‐PP, C) 6% Ca‐PP, D) 10% Ca‐PP, and E) 12.5% Ca‐PP. The interface between the heterotopic bone and the CaP material—white dotted line. Orientation of the osteocytes—yellow arrowheads. Scale bars = 50 µm.

The second region of interest (ROI) assessed with Raman spectroscopy was the CaP material in close proximity to the bone interface. At both 12‐ and 52 weeks in vivo, the CaP material at the interface underwent a substantial transformation into CHAp independent of the initial amount of Ca‐PP (**Figure** **3**). This likely occurred via the transformation of the monetite and β‐TCP phases, as they were no longer detectable in the Raman spectra (**Figure** **4**). At 52 weeks, from the original components of our CaP material, only the Ca‐PP phase remained.

### 2.4 Qualitative Histology of Heterotopic Bone

Irrespective of the composition of the CaP construct, at 12 weeks in vivo, a thin layer of heterotopic bone was found covering the material surface (Figure 1A). Toluidine blue staining of undecalcified histological sections revealed that at 12 weeks, bone followed the contours of the CaP material that had undergone partial degradation (Figure 4). In all compositions, the osteocytes were aligned parallel to the bone‐material interface, and the lamellar arrangement of bone was apparent from the striated appearance of the extracellular matrix (Figure 4A–E).

**Figure 5:**
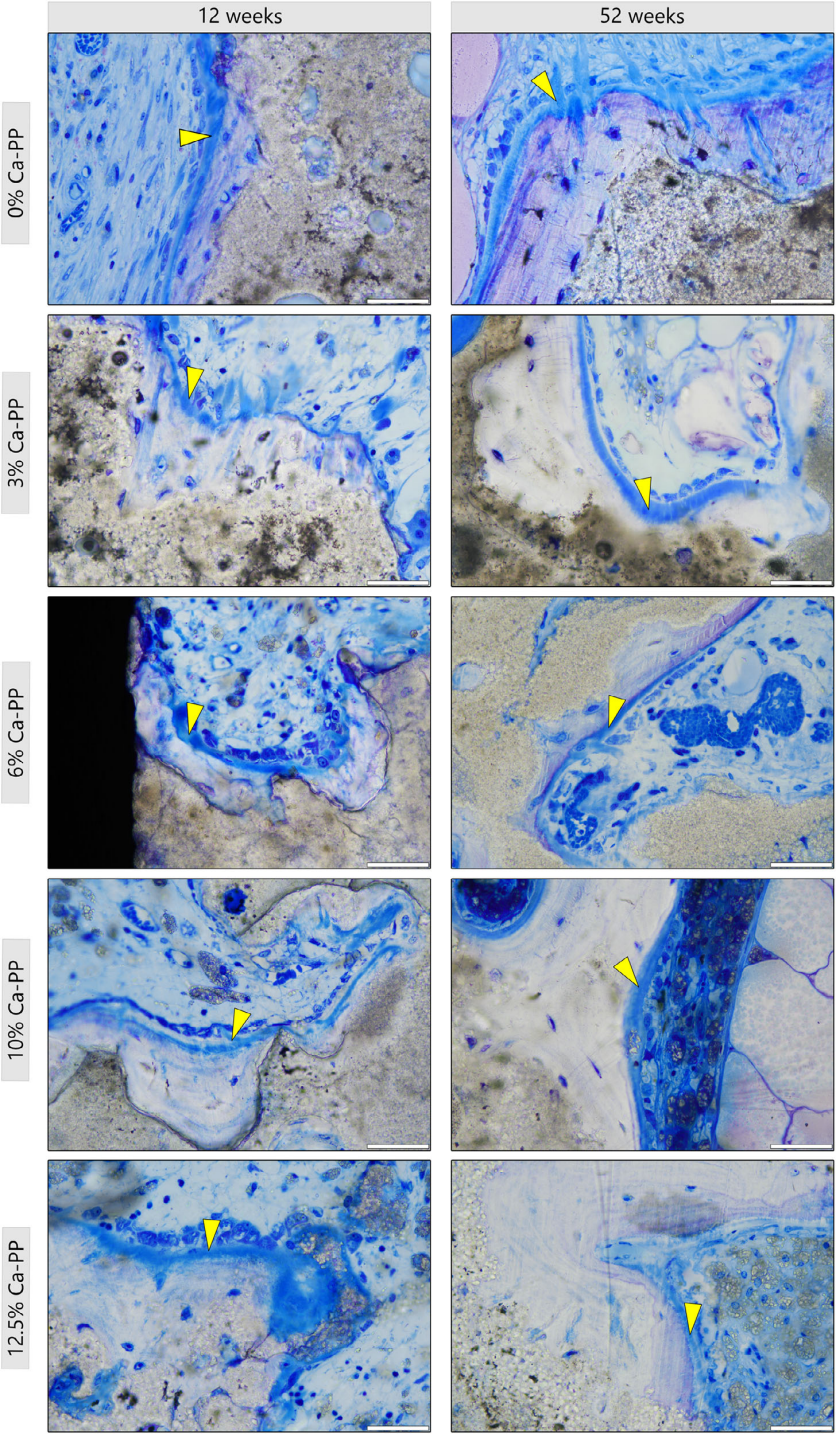
Presence of osteoid at 12‐ and 52 weeks in vivo. Representative, undecalcified, toluidine blue stained histological sections showing non‐mineralized bone matrix, osteoid (yellow arrowheads), associated with all CaP material compositions. Scale bars = 50 µm.

Osteoid, bone matrix that has not yet undergone mineralization, was identified in all compositions at 12‐ and 52 weeks (**Figure** **5**). At the surface of this newly formed bone matrix, osteoblasts were frequently observed, confirming ongoing bone formation. While osteoid areas were found at both time points, the incidence of these areas was reduced at 52 weeks.

**Figure 6:**
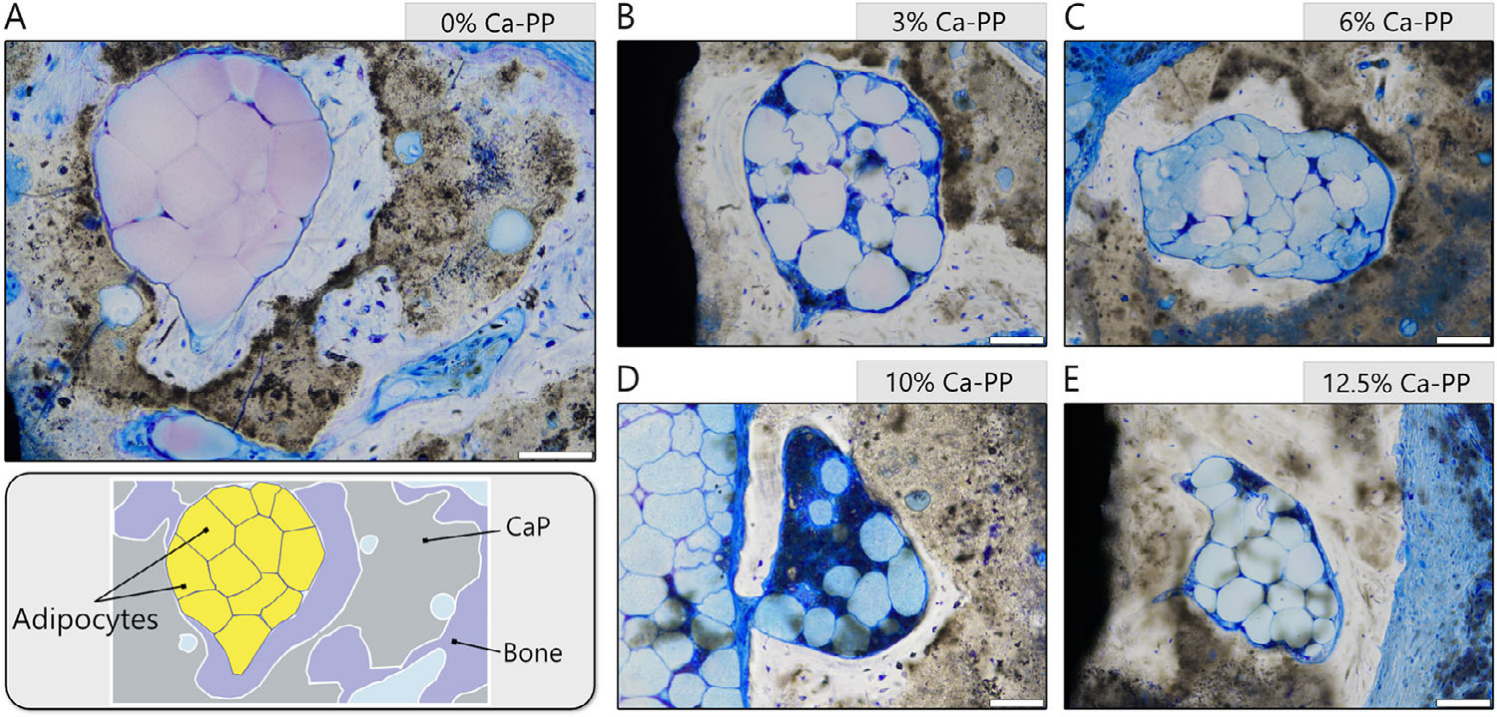
Presence of adipose cells at 52 weeks in vivo. Representative, undecalcified, toluidine blue stained histological sections showing the presence of the adipocytes in larger islands of heterotopic bone for: A) 0% Ca‐PP, B) 3% Ca‐PP, C) 6% Ca‐PP, D) 10% Ca‐PP, and E) 12.5% Ca‐PP at 52 weeks. Scale bars = 100 µm.

At 52 weeks in vivo, larger islands of bone were found. Moreover, large fat cells, adipocytes, were found in all investigated compositions (**Figure** **6**A–E). These cells were observed not only in the soft tissue of the inter‐tile space but also in large islands of bone within the remaining CaP material bulk. Seemingly, these adipocytes occupied areas in which heterotopic bone has undergone remodeling. Here, the lamellar bone structure was more obvious, while the material interface was more clearly demarcated.

**Figure 7:**
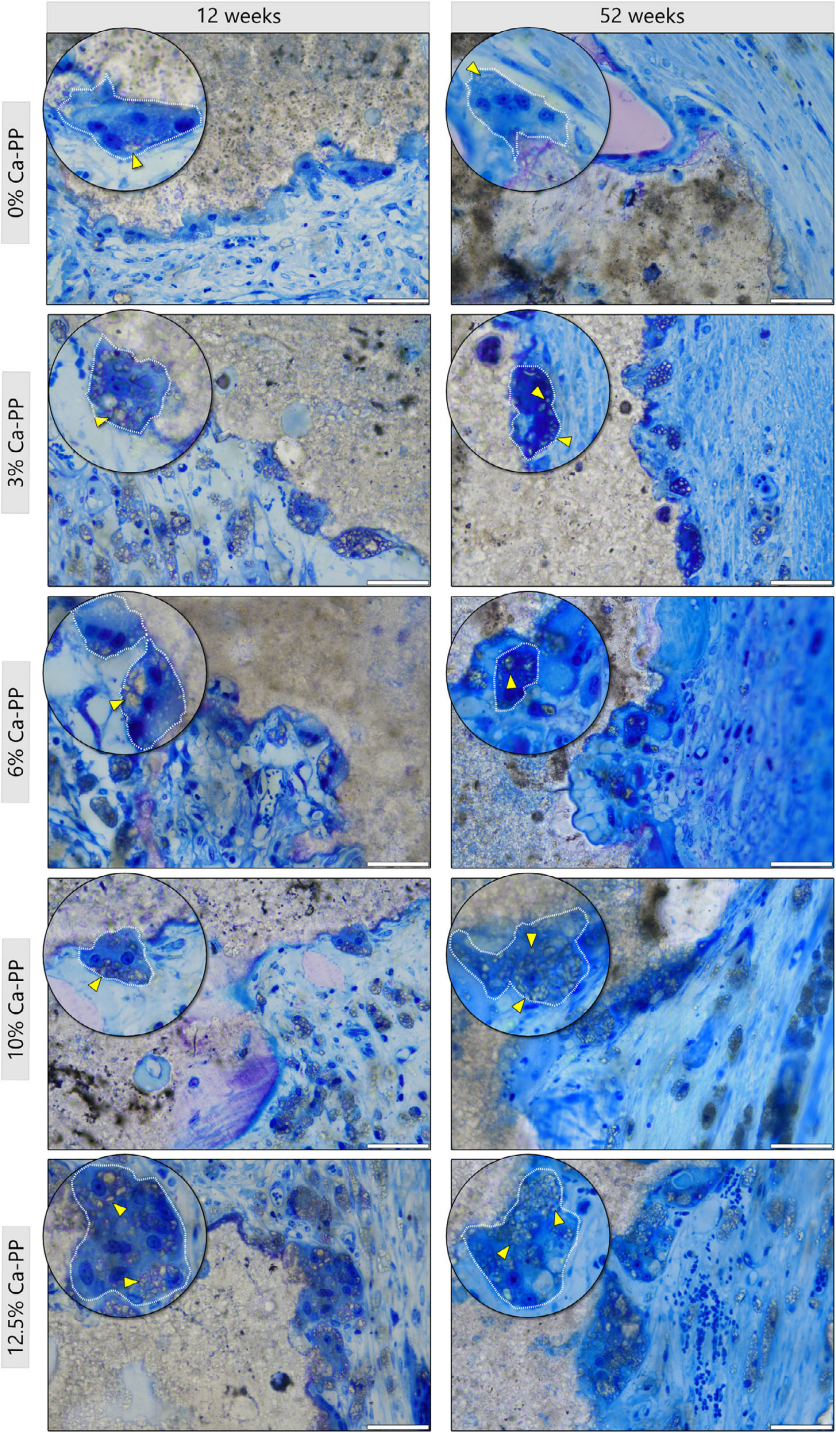
CaP is actively removed at 12‐ and 52 weeks in vivo. Representative, undecalcified, toluidine blue stained histological sections showing multinucleated cells near all CaP material compositions. Large particles of CaP material (yellow arrowheads) within the intracellular space. Scale bars = 50 µm.

The CaP material underwent significant degradation in vivo, which appeared to be dependent not only on the exposure of the material to the surrounding environment (i.e., interstitial fluid) but also facilitated by cells. Multinucleated cells were often observed at the surface of the CaP material, with CaP particles clearly visible intracellularly at 12‐ and 52 weeks (**Figure** **7**; Figure S14, Supporting Information). At 52 weeks, few multinucleated cells were observed in association with CaP constructs containing 0% Ca‐PP, and in all other compositions, their numbers were reduced compared with 12 weeks in vivo.

### 2.5 Chemical Characterization of Heterotopic Bone

**Figure 8:**
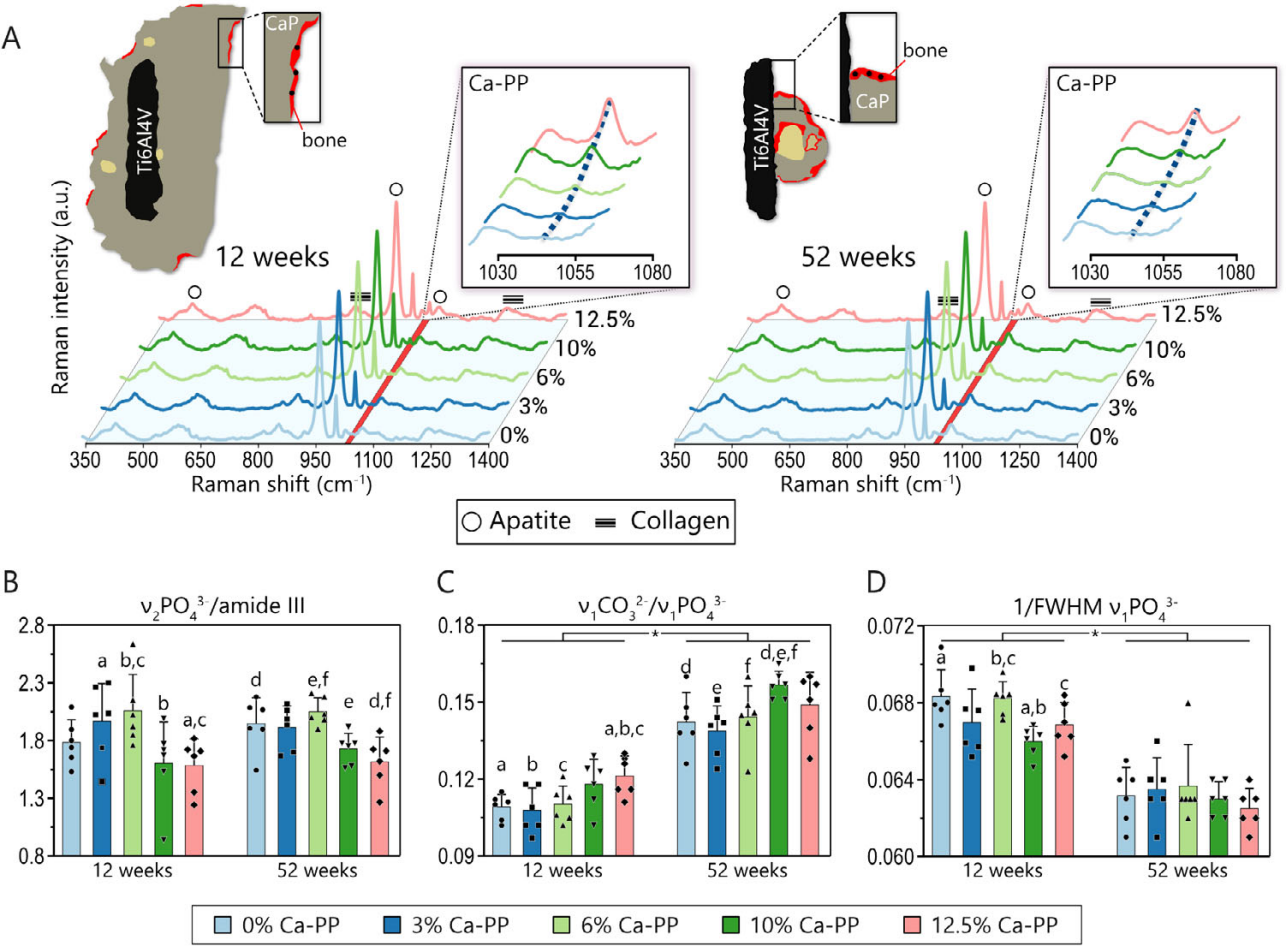
Chemical composition of heterotopic bone. A) Top: Schematic representation of CaP constructs at 12‐ and 52 weeks in vivo and locations of Raman spectroscopy point measurements (black dots) in heterotopic bone at 12‐ and 52 weeks. Averaged Raman spectra (*n* = 6) for respective compositions (0–12.5% Ca‐PP). Characteristic spectral features of bone: apatite (≈960 cm⁻^1^), ν_1_CO_3_^2^⁻ (≈1070 cm⁻^1^), amide III (1240–1270 cm⁻^1^), proline and hydroxyproline (850–873 cm⁻^1^). Insets show 1030–1080 cm⁻^1^ range of Raman spectra and the appearance of the Ca‐PP peak at 1043 cm⁻^1^. Extracellular matrix characterization: B) mineral‐to‐matrix ratio as the integral area ratio of ν_2_PO_4_^3^⁻ (420–470 cm⁻^1^) and amide III (1240–1270 cm⁻^1^) bands, C) carbonate‐to‐phosphate ratio as the intensity ratio of ν_1_CO_3_^2^⁻ (≈1070 cm⁻^1^) and ν_1_PO_4_^3^⁻ (≈960 cm⁻^1^) peaks, and D) mineral crystallinity as the inverse full‐width‐at‐half maximum (FWHM) of the ν_1_PO_4_^3^⁻ peak (≈960 cm⁻^1^). The same letters indicate a significant difference between the two compositions. The asterisk indicates a significant difference between respective compositions at two‐time points.

The chemical composition of heterotopic bone was investigated using Raman spectroscopy. At both 12‐ and 52 weeks, characteristic spectral features of the mineral (i.e., CHAp) and organic phases (i.e., amide III, proline, hydroxyproline) of bone were identified. The Raman spectra were comparable across the various CaP compositions at 12‐ and 52 weeks (**Figure** **8**A). Notably, the Ca‐PP peak was detected in the Raman spectra of heterotopic bone, with the ≈1043 cm⁻^1^ peak appearing at both 12‐ and 52 weeks (Figure 8A).

The degree of mineralization, as indicated by the mineral‐to‐matrix ratio, was mostly comparable between the CaP compositions, with an average ν_2_PO_4_^3^⁻/amide III ratio of 1.8 ± 0.3 and 1.85 ± 0.2 at 12‐ and 52 weeks, respectively. Generally, the degree of mineralization was higher in response to constructs containing lower amounts of Ca‐PP (0, 3, and 6%) (**Table** **1**).

**Table 1:** Raman metrics of heterotopic bone composition at 12‐ and 52 weeks in vivo.

Composition	*12 weeks*			*52 weeks*		
	ν_2_PO_4_^3^⁻/ amide III	ν_1_CO_3_^2^⁻/ ν_1_PO_4_^3^⁻	1/FWHM ν_1_PO_4_^3^⁻	ν_2_PO_4_^3^⁻/ amide III	ν_1_CO_3_^2^⁻/ ν_1_PO_4_^3^⁻	1/FWHM ν_1_PO_4_^3^⁻
*0% Ca‐PP*	1.79 ± 0.20	0.11 ± 0.01	0.068 ± 0.001	1.95 ± 0.23	0.14 ± 0.01	0.063 ± 0.001
*3% Ca‐PP*	1.97 ± 0.33	0.11 ± 0.01	0.067 ± 0.002	1.91 ± 0.19	0.14 ± 0.01	0.064 ± 0.002
*6% Ca‐PP*	2.06 ± 0.31	0.11 ± 0.01	0.068 ± 0.001	2.05 ± 0.12	0.14 ± 0.01	0.064 ± 0.002
*10% Ca‐PP*	1.60 ± 0.36	0.12 ± 0.01	0.066 ± 0.001	1.73 ± 0.13	0.16 ± 0.01	0.063 ± 0.001
*12.5% Ca‐PP*	1.59 ± 0.23	0.12 ± 0.01	0.067 ± 0.001	1.62 ± 0.21	0.15 ± 0.01	0.063 ± 0.001

At 12 weeks, the mineral‐to‐matrix ratio was highest for 6% Ca‐PP, with significant differences in comparison with 10% and 12.5% Ca‐PP (*p* < 0.05 and *p* < 0.01, respectively) (Figure 8B). The next highest mineral‐to‐matrix ratio was seen for 3% Ca‐PP (*p* < 0.05 vs. 12.5% Ca‐PP) (Table 1). At 52 weeks, the mineral‐to‐matrix ratio was also highest for 6% Ca‐PP (*p* < 0.01 vs. 10%‐ and 12.5% Ca‐PP), while the second highest ratio was seen for 0% Ca‐PP (*p* < 0.05 vs. 12.5% Ca‐PP). At both time points, the lowest mineral‐to‐matrix ratio was observed for 12.5% Ca‐PP.

For all compositions, the carbonate‐to‐phosphate ratio significantly increased with time. This increase reflects B‐type carbonate substitution (replacement of PO_4_^3^⁻ by CO_3_^2^⁻) in the apatite structure, leading to changes in size and increased solubility of the mineral.^[^ ^20^ ^]^ We found significantly lower carbonate‐to‐phosphate ratios with constructs containing low amounts of Ca‐PP at both time points (Table 1). At 12 weeks, the carbonate‐to‐phosphate ratio for 12.5% Ca‐PP was significantly higher than 0‐, 3‐, and 6% Ca‐PP (*p* < 0.05). At 52 weeks, the highest carbonate‐to‐phosphate ratio was found for 10% Ca‐PP, which was significantly higher than 0‐, 3‐, and 6% Ca‐PP (*p* < 0.05 vs. 0‐ and 6% Ca‐PP, and *p* < 0.01 vs. 3% Ca‐PP) (Figure 8C).

The mineral crystallinity (1/FWHM ν_1_PO_4_^3^⁻) decreased from 12‐ to 52 weeks in vivo (Table 1). At 12 weeks, significant intergroup differences could be calculated. The mineral crystallinity was significantly higher for 6% Ca‐PP than 10‐ and 12.5% Ca‐PP (*p* < 0.01) and for 0% Ca‐PP than 10% Ca‐PP (*p* < 0.01) (Figure 8D).

## 3 Discussion

Despite advances in bone repair and reconstruction, large bone defects from trauma or tumor resections remain a major challenge in maxillofacial and orthopedic surgery. The heterotopic bone formation reported here occurs in response to an osteoinductive material and should not be mistaken for pathological calcification,^[^ ^21^ ^]^ which is noted in soft tissues as a complication of trauma or surgery.^[^ ^22^ ^]^ In this work, we have examined the osteoinductive properties of a series of multi‐component CaP materials implanted in a subcutaneous sheep model. Different compositions were prepared with fixed amounts of monetite and β‐TCP at a 10:1 ratio and varying Ca‐PP content (0–12.5%). CaP materials composed of monetite, β‐TCP, and Ca‐PP are deemed safe for clinical use in the repair of large cranial defects.^[^ ^19^, ^23^ ^]^ However, they are not intended for high load‐bearing applications and may require reinforcement such as with a titanium mesh to achieve stiffness and strength comparable to that of the native skull bone.^[^ ^24^ ^]^

The combination of monetite and β‐TCP is known to support heterotopic ossification (i.e., osteoinduction), on the other hand, inorganic pyrophosphate ions have long been recognized as inhibitors of apatite precipitation and mineralization because of their role in preventing crystal nucleation.^[^ ^25^ ^]^ We found that the incorporation of Ca‐PP in the multi‐component CaP material had minimal impact on heterotopic bone formation. After 12‐ and 52 weeks in vivo, heterotopic bone was consistently detected across all compositions, regardless of Ca‐PP content, though higher amounts compromised *de novo* bone quality. Furthermore, the mineral component of the heterotopic bone was carbonated apatite, as expected in natural bone.^[^ ^26^ ^]^ Moreover, we observed no heterotopic bone formation in response to Ti6Al4V ELI implants, highlighting the osteoinductive properties of the tested multi‐component CaP material.

It has been reported that the addition of a high amount of Ca‐PP (i.e., 28 wt.%) to brushite cements enhances bone formation in a sheep bone‐defect model.^[^ ^15^ ^]^ In this example, poor interfacial adhesion between the Ca‐PP particles and brushite probably lowers the durability of the material, which facilitates the creation and dispersal of material fragments. This process thereby increases the available surface area for further material degradation, favoring osteoconduction and a larger de novo bone area.^[^ ^15^ ^]^ Here, we did not observe a similar osteogenic effect of Ca‐PP, as the amount of Ca‐PP added (0–12.5%) did not influence the extent of CaP material degradation. This is further highlighted by a time‐dependent increase in B.Ar from 12‐ to 52 weeks in vivo that reached statical significance only upon normalization to CaP.Ar. The increase in B.Ar (with respect to CaP.Ar) is attributable to the substantial CaP material degradation by 52 weeks in vivo, with a direct correlation between the B.Ar and CaP.Ar.

It is generally assumed that when a bone repair biomaterial degrades, the resulting voids allow the in‐growth of bone, whereby the amount of remaining implant material is inversely correlated with the area (or volume) occupied by newly formed bone. A characteristic feature of bone regeneration within bone defects using biomaterials is that while occupying an enclosed space, any newly formed bone is *attached* to both the implant material and the native bone. In contrast, without confinement by the native bone, such as in a soft tissue implantation site, the newly formed bone is primarily attached to the implant material. Upon degradation, fragments of the implant material are *cleared* (or removed) from the local site along with the newly formed attached bone. In other words, degradation of the implant material is expected to contribute to a partial loss of the newly formed bone. Therefore, the amount of heterotopic bone amenable to quantitative analysis is dependent on the amount of the remaining implant material.

One of the desirable properties of bone substitute materials is a degradation rate that matches that of new bone formation.^[^ ^27^ ^]^ Therefore, understanding the degradation process of the investigated material is of special importance. Following implantation, all CaP constructs underwent substantial degradation in vivo, the extent of which was unrelated to the Ca‐PP content. At least two mechanisms contribute to this process: *i*) passive material degradation driven by factors such as mechanical stresses from the physiological environment and *ii*) active material degradation involving cells. Solubility is a key factor in the loss of mass of CaP materials with low porosity during in vivo degradation.^[^ ^28^ ^]^ Monetite, which constitutes ≈80–91% in this series of compositions, may be the main driving force behind the degradation of the CaP material between 12‐ and 52 weeks in vivo, given its greater solubility than that of both β‐TCP and Ca‐PP at neutral pH.^[^ ^29^ ^]^

On the surface and in close proximity to the implanted constructs, multinucleated cells containing aggregates of CaP fragments (also referred to as “*material‐filled macrophages*”^[^ ^30^ ^]^) were discernible in the intracellular space, both at 12‐ and 52 weeks. In similar Ca‐PP containing CaPs, this intracellular debris has been shown to consist exclusively of Ca‐PP particles.^[^ ^19^, ^31^ ^]^ In our work, we did not find that the extent of material bulk degradation is changed by increasing the amount of Ca‐PP, although higher amounts of Ca‐PP appear to exacerbate the phagocytosis of the CaP material. We noted that the area occupied by multinucleated cells with intracellular CaP debris increased with the amount of added Ca‐PP from 3% to 12.5%, whereas with 0% Ca‐PP, these cells were scarcely found. A similar observation was made in a subcutaneous rat model where higher amounts of phagocytized microparticles were found with four β‐TCP implants doped with increasing amounts of Ca‐PP (0.5–10 wt.%). A higher amount of Ca‐PP was associated with the presence of inflammatory cells at 4 weeks following implantation.^[^ ^32^ ^]^ Here, we did not observe an increase in inflammation during visual inspection of the implantation site at sample retrieval, and upon histological investigation, the increased presence of multinucleated cells corresponded with material degradation. The lack of definitive information on cellular response to CaP materials during the early healing stage (i.e., <12 weeks) may be considered a limitation. High concentrations of pyrophosphate prolong monocyte survival and stimulate pro‐osteogenic gene expression in mesenchymal stem cells in vitro.^[^ ^33^ ^]^ However, this effect of pyrophosphate on mesenchymal stem cells is likely indirect and mediated through monocyte‐derived factors, highlighting the complex crosstalk between immune response and stem cells in bone formation, reinforcing the need for in vivo studies which provide a more complex biological system. Future studies should incorporate immunohistochemical and more advanced spatial omics approaches (e.g., spatial transcriptomics and proteomics) to further dissect the immunomodulatory and osteoinductive effects of CaP materials.

We also found that the CaP material underwent substantial chemical transformation in vivo. Depending on the pH and composition of the microenvironment, CaPs can transform from one phase to another.^[^ ^34^ ^]^ Here, after 12 weeks in vivo, Raman spectroscopy revealed the presence of apatite in addition to the constituent phases of the multi‐component CaP material. This transformation is attributed primarily to the dissolution of monetite, as β‐TCP is insoluble in physiological conditions. However, the solubility of β‐TCP increases at acidic pH, leaving cell‐mediated resorption as a main mode of dissolution.^[^ ^11^ ^]^ After 52 weeks, a near‐complete transformation was noted at the CaP material‐bone interface, with apatite being the predominant CaP phase detected. However, the presence of Ca‐PP peaks within the Raman spectra points toward low solubility of this phase in vivo and highlights the importance of phagocytosis as a primary means of Ca‐PP removal in non‐osseous sites.

In areas of the CaP material located farthest from the bone interface, we found that the transformation to apatite was less pronounced at 12 weeks in vivo, confirming that exposure to cellular activity increases the chemical transformation of the material. Nevertheless, at 52 weeks in vivo, the material had undergone considerable transformation at these distant sites. In the compositions with 0‐ and 6% Ca‐PP, the monetite phase was no longer detectable in the averaged Raman spectra, even though it could be observed in the individual point measurements. In contrast, in the compositions with 3‐, 10‐, and 12.5% Ca‐PP, the averaged Raman spectra showed the presence of all initial phases (i.e., monetite, β‐TCP, and Ca‐PP). As the selection of ROIs was performed using backscattered electron scanning electron microscopy (BSE‐SEM), the obtained information is limited to component distribution (i.e., CaP, bone, and surrounding tissues) at the sample surface. It is possible that the greater extent of degradation observed in compositions with 0‐ and 6% Ca‐PP compositions at 52 weeks was due to the sampled sites being in closer proximity to bone and/or interstitial fluid than intended, introducing an unintentional bias into our measurements.

Several questions regarding the long‐term fate of the CaP material remain, specifically: *i*) what happens with the phagocytized CaP material during cell mitosis or apoptosis, *ii*) will the observed multinucleated cells still be present in the subcutaneous pocket once the remaining bulk CaP material has been degraded, and *iii*) what is the fate of the already phagocytized CaP material if it cannot be resorbed? Non‐degradable microparticles internalized through phagocytosis, can be divided between daughter cells during mitosis, and if apoptosis occurs, the released particles may be taken up by other macrophages.^[^ ^35^ ^]^ It remains to be seen if intracellular CaP debris can be resorbed with prolonged exposure to the acidic environment in the phagosome or if a similar scenario could apply here. It is expected that the ongoing degradation of the implanted CaP material will continuously provide new debris for phagocytosis. During degradation in vivo, BCPs release HAp particles that are phagocytized by macrophages but are not effectively degraded, leading to local tissue inflammation and cell damage.^[^ ^36^ ^]^ It stands that if phagocytized CaP material particles are resistant to dissolution and degradation, they will likely contribute to cellular stress and apoptosis, triggering the release of the inflammation mediators, inducing repeated phagocytosis, and ultimately leading to fibrous encapsulation of the particles in the subcutaneous pocket.

Beyond the effect on degradation of the CaP material, we investigated the impact of Ca‐PP on heterotopic bone formation and quality. At 12 weeks, heterotopic bone formation was restricted mainly to the outer regions of the implants and was rarely observed within the bulk of CaP material. This is most likely caused by the low interconnected porosity of our material. Here, the heterotopic bone formed through intramembranous ossification with little evidence of an intermediate cartilage. At 12 weeks in vivo, heterotopic bone followed the contours of the partially degraded CaP material and had a lamellar‐like organization. The lamellar‐like appearance is interpreted as coordinated bone formation activity resulting from the initial organization of osteoblasts on the surface of the substrate (e.g., CaP material), which plays a critical role in the formation of ordered tissues.^[^ ^37^ ^]^ The bone continuously formed on the material surface. In toluidine blue‐stained histological sections, non‐mineralized bone tissue (i.e., osteoid) was found across all compositions at both 12‐ and 52 weeks in vivo. In the majority of cases, the osteoid was associated with polarized surface osteoblasts. However, the incidence of such observations was reduced at 52 weeks for all CaP constructs regardless of the amount of Ca‐PP. This sustained bone formation is attributed to the continuous degradation of the CaP material, including the development of cracks within the material, particle dispersal, and release of Ca^2+^ and PO_4_^3^⁻.

After 52 weeks in vivo, large islands of bone were found in all compositions. This bone occupied both the surface concavities and large pores within the degraded CaP material. The most striking observation in all compositions was that of large adipocytes in bone areas that had undergone a remodeling process, creating compartments resembling bone marrow. It is well‐documented that disuse osteopenia, i.e., bone loss due to local skeletal unloading, is associated with the accumulation of fat in the bone marrow.^[^ ^38^ ^]^ We presume that the accumulation of adipocytes in heterotopic bone in our study is caused by the lack of mechanical loading within the subcutaneous pocket. During normal bone remodeling within the skeletal system, the amount of resorbed bone is in net balance with the amount of newly formed bone to ensure the structural integrity of the tissue.^[^ ^39^ ^]^ However, here, it appears that once the strong pro‐osteogenic microenvironment generated by the material‐induced immune response subsided, the remodeling processes that took place favored the differentiation of mesenchymal cells into adipocytes. In another study, heterotopic bone formed in response to HAp and BCP implants and bone‐like tissue in β‐TCP implants were found as long as 2.5 years in vivo after implantation in the dorsal muscles of dogs.^[^ ^40^ ^]^ The bone exhibited clear signs of remodeling and contained a bone marrow compartment, consistent with our findings. While the presence of heterotopic bone in response to the implanted materials confirms that long‐term survival of heterotopic bone is possible, it is contingent on the continued availability of the CaP material at a non‐osseous site.

The presence of Ca‐PP did not affect the composition of the heterotopic bone formed at either 12‐ or 52 weeks in vivo, with typical organic and mineral spectral features of extracellular bone matrix present in Raman spectra. However, we observed the presence of the Ca‐PP peak (≈1043 cm⁻^1^) in the Raman spectra of the extracellular bone matrix. In a previous report, this Ca‐PP peak was found in the Raman spectra of extraskeletal bone formed in response to CaP constructs with a similar composition, which was ascribed to the presence of discrete Ca‐PP particles embedded within the bone matrix.^[^ ^19b^ ^]^ Here, we noted a positive relationship between the intensity of the Ca‐PP signal in the Raman spectra and the amount of Ca‐PP in the initial CaP mixture, suggesting that higher initial amounts of Ca‐PP lead to greater incorporation of discrete particles of Ca‐PP within the heterotopic bone matrix. Still, our findings suggest that the inclusion of Ca‐PP in an osteoinductive material may negatively affect the level of mineralization of *de novo* bone. At both 12‐ and 52 weeks, mineralization levels of heterotopic bone were the lowest in compositions with the highest amounts of Ca‐PP (i.e., 10‐ and 12.5%). Although the mineral‐to‐matrix ratio, as well as the carbonate‐to‐phosphate ratio, generally increase with tissue age, here, the mineral content did not increase with time from 12‐ to 52 weeks.^[^ ^41^ ^]^ While this could be due to a process where CaP material is continuously degraded and *de novo* bone is consequently formed, the presence of larger islands of bone tissue with osteonal organization and clear signs of remodeling in the heterotopic bone at 52 weeks render such a scenario unlikely. Both these observations suggest a more “mature” bone tissue at 52 weeks, contrasting with the lamellar‐like bone on the surface of the constructs at 12 weeks. An alternative explanation for the comparable levels of mineralization at the two time points is that amounts of mineral and collagen increased proportionally within the extracellular bone matrix of heterotopic bone. This notion is supported by the higher carbonate‐to‐phosphate ratio of bone found in all compositions at 52 weeks (*p* < 0.01). We observed a gradual increase in B‐type carbonate substitution in the heterotopic bone from 0‐ to 12.5% Ca‐PP, with the highest values measured for 12.5‐ and 10% Ca‐PP at 12‐ and 52 weeks, respectively. It appears that higher amounts of Ca‐PP within the extracellular bone matrix resulted in delayed bone formation and/or bone remodeling. Furthermore, CO_3_^2^⁻ incorporation into the apatite lattice negatively correlated with mineral crystallinity (1/FWHM ν_1_PO_4_^3^⁻). Generally, substitutions in the apatite lattice structure are indicative of changes in the perfection and size of crystallites and are negatively correlated with mineral crystallinity.^[^ ^42^ ^]^ However, large changes in CO_3_^2^⁻ content do not always result in significant changes in the FWHM of ν_1_PO_4_^3^⁻.^[^ ^43^ ^]^ At 12 weeks, the lowest mineral crystallinity was observed in the bone that formed in response to constructs with the highest amounts of Ca‐PP (10‐ and 12.5%), whereas at 52 weeks, the same trend was not observed.

## 4 Conclusion

In summary, the present study was designed to determine the impact of a multi‐component CaP material comprising 0‐ to 12.5% Ca‐PP on heterotopic bone formation and the fate of the material in vivo. We showed that the presence of Ca‐PP does not hinder heterotopic bone formation and has a negligible role in the degradation of the CaP material. However, larger amounts of Ca‐PP negatively influenced the level of heterotopic bone mineralization. The incorporation of Ca‐PP within the bone extracellular matrix may impair the mechanical properties of bone that were not explored in this work. Lastly, we confirmed that Ca‐PP has low solubility in vivo, with phagocytosis being the main mode of removal, although the long‐term impact of phagocytized Ca‐PP and the fate of phagocytizing multinucleated cells remains to be determined. Phagocytized Ca‐PP could accumulate in the surrounding tissues long after the degradation of the implanted material. Notably, Ca‐PP is among the main impurities associated with β‐TCP synthesis, with the International Organization for Standardization (ISO) standard (13175‐3:2012) describing any β‐TCP product with up to 5 wt.% of foreign phase as pure.^[^ ^44^ ^]^ Long‐term in vivo studies are needed to determine the fate of biomaterials containing Ca‐PP, including the fate of degradation products, as well as the influence of Ca‐PP on bulk mechanical properties of *de novo* bone.

## 5 Experimental Section

### Experimental Setup

**Figure 9:**
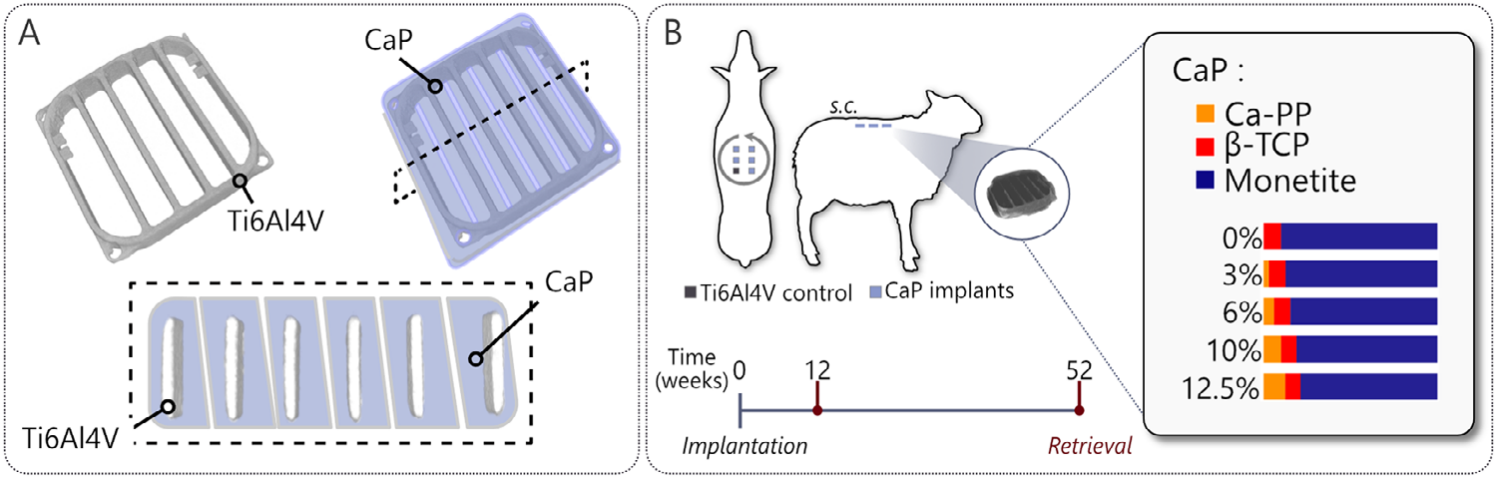
Experimental setup. A) Calcium phosphate (CaP) implant design with an embedded Ti6Al4V ELI frame. B) Subcutaneous placement (s.c.) of five experimental CaP constructs and one control construct (Ti6Al4V only) in 12 adult female sheep (*n* = 6 sheep/time point). Study timeline and CaP composition.

The multi‐component CaP material was made from monetite‐forming calcium‐based precursor powder comprising monocalcium phosphate monohydrate [Ca(H_2_PO_4_)_2_•H_2_O, MCPM; Alfa Aesar, Thermo Fisher] and beta‐tricalcium phosphate [β‐Ca_3_(PO_4_)_2_, β‐TCP; Sigma‐Aldrich] with the addition of dicalcium pyrophosphate (Ca_2_P_2_O_7_, Ca‐PP; Sigma‐Aldrich) and glycerol (Sigma–Aldrich) as described previously.^[^ ^19^, ^45^ ^]^ To investigate the influence of Ca‐PP on heterotopic bone formation and CaP degradation, five material compositions were made (Table S1, Supporting Information) by maintaining a constant MCPM to β‐TCP ratio and adding an increasing amount of Ca‐PP powder (0, 3, 6, 10, and 12.5%). The CaP‐glycerol paste was molded over 3D printed Ti6Al4V ELI frames (6 × 18 × 18 mm) and allowed to set overnight in sterile water. The constructs were removed from the molds and placed in sterile water for an additional 48 h to remove excess glycerol. The final constructs were cut to form six connecting CaP tiles of varying sizes (**Figure** **9**A). Control implants were made entirely of 3D printed Ti6Al4V ELI framed mesh with dimensions comparable to those of the experimental CaP implants. All implants were steam sterilized in an autoclave at 121 °C for 20 min.

### Animal Experiment and Sample Processing

Twelve adult, skeletally mature female sheep (*Ovis aries*) received subcutaneous implants. The implants were placed at a non‐osseous site between the skin and the muscle of the sheep to allow us to investigate the true osteoinductive potential of the CaP material. Anesthesia in the animals was induced via intravenous propofol injection and maintained with isoflurane inhalation. Under anesthesia, the selected surgical site was clipped free of wool, cleaned with povidone‐iodine, and draped to maintain a sterile field. Three incisions were made on each side of the dorsal area, parallel to the vertebral column, to create subcutaneous pockets with blunt dissection. Each sheep received five different CaP constructs consisting of monetite, β‐TCP, with an increasing amount of Ca‐PP (0, 3, 6, 10, and 12.5% Ca‐PP), as well as one Ti6Al4V implant as control. The exact location of the six implants was determined according to a randomization scheme (Figure 9B). To prevent movement under the skin, the implants were attached to the back muscles of the animals by a surgical thread guided through lateral hoops built into the Ti6Al4V ELI frame. The animals received an intramuscular injection of buprenorphine each day for two days after the surgery. The anti‐inflammatory drug, flunixin, was given for 7 days post‐surgery, and antibiotics, amoxicillin and enrofloxacin, for 6 weeks post‐surgery. After 12‐ and 52 weeks (*n* = 6 sheep/time point), the animals were euthanized via pentobarbital intravenous injection. Implants were dissected *en bloc* with the surrounding soft tissue, fixed in 10% neutral buffered formalin, dehydrated in ethanol, and resin embedded (LR White, London Resin Co. Ltd., UK). The animal experiments were approved by the Ministry of National Education, Higher Education and Research (NAMSA, Chasse‐sur‐Rhône, France; Approval nr. 01139.2).

### Histology and Histomorphometry

Undecalcified, toluidine blue and Van Gieson‐stained histological sections (≈40 µm thick) were prepared from bisected, resin‐embedded blocks. Areas where collagen was present stain positively with Van Gieson's stain, and therefore, collagenous sites (i.e., heterotopic bone) and non‐collagenous sites (i.e., CaP) can be readily distinguished. Toluidine blue stain was used in a second set of histological sections to determine the presence and involvement of different cellular components. Brightfield imaging was performed using a Nikon Eclipse E600 optical microscope (Nikon Ltd., Tokyo, Japan). Van Gieson‐stained sections were imaged using 10× magnification and used to measure the heterotopic B.Ar and CaP.Ar present in the sections. Area measurements were made in ImageJ (imagej.nih.gov/ij) by manually demarcating the areas of interest—heterotopic bone and the CaP material.

### Raman Spectroscopy and Backscattered Electron Scanning Electron Microscopy (BSE‐SEM)

The remaining halves of the resin‐embedded blocks were wetly polished using 400–4000 grit silicon carbide (SiC) paper, with the final polishing step performed with absolute ethanol. Initial identification of ROIs was made using BSE‐SEM in a Quanta 200 environmental SEM (FEI Company, The Netherlands) operated at 20 kV acceleration voltage, 1 Torr water vapor pressure, and 10 mm working distance (Figures S1–S10, Supporting Information). Three distinct ROIs were selected: *i*) heterotopically formed bone, *ii*) CaP located 50–100 µm from the bone interface, and *iii*) CaP areas furthest from the bone interface, typically within 50 µm from the Ti6Al4V ELI frame. Additionally, energy dispersive X‐ray spectroscopy (EDX) maps of phagocytized CaP material (264 × 214 µm) were made in the inter‐tile area of two representative samples, one for each time point, at 15 kV, 1 Torr water vapor pressure, and 10 mm working distance. For chemical composition analysis of the CaP material and heterotopic bone, Raman spectroscopy was performed using a confocal Raman microscope (Renishaw inVia Qontor, Renishaw plc. Wotton under Edge, UK) equipped with a 785 nm wavelength laser and Live‐Track focus‐tracking technology for enhanced signal stability.^[^ ^46^ ^]^ The laser was focused on the sample surface through a 100x/0.9 NA objective, and the Raman scattered light was collected using a Peltier‐cooled charge‐coupled device deep depletion near‐infrared enhanced detector behind a 1200 g mm⁻^1^ grating. The laser power at the sample was ≈10 mW. On each sample (*n* = 6), six point measurements were obtained at 2 s integration time and 5 accumulations in each ROI. Background subtraction and cosmic ray removal were performed in Spectragryph V1.2.16.1 (Figure S11, Supporting Information).^[^ ^47^ ^]^ In heterotopic bone, mineral crystallinity corresponds to the inverse full‐width at half‐maximum (1/FWHM) of ν_1_PO_4_^3^⁻ peak (≈960 cm⁻^1^). The carbonate‐to‐phosphate ratio is the intensity ratio between the ν_1_CO_3_^2^⁻ (≈1070 cm⁻^1^) and ν_1_PO_4_^3^⁻ (≈960 cm⁻^1^) peaks. The mineral‐to‐matrix ratio is the integral area ratio between the ν_2_PO_4_^3^⁻ (420–470 cm⁻^1^) and the amide III (1240–1270 cm⁻^1^) bands.

### Statistical Analysis

Statistical analyses were performed using GraphPad Prism v10.0.0 (GraphPad Software, USA). The non‐parametric Mann–Whitney U test was used for comparisons between groups, and *p* values <0.05 were considered statistically significant. Mean values ± standard deviations (SD) are presented. Spearman correlation analysis was performed between the B.Ar and CaP.Ar. Based on the value of the Spearman's rank correlation coefficient, the strength of the correlation was interpreted as: little if any correlation (0.00–0.30), low (0.30–0.50), high (0.70–0.90), and very high (0.9–1.00).

## Conflict of Interest

P.T. reports a relationship with OssDsign AB that includes: equity or stocks. T.E. and H.E. report a relationship with OssDsign AB that includes: consulting or advisory and equity or stocks. T.E. has patent #US9220597B232 licensed to OssDsign AB. H.E. has patent #US9220597B232 licensed to OssDsign AB.

## Author Contributions

M.J. contributed to methodology, investigation, visualization, and writing—original draft. I.Å. contributed to methodology. O.O. contributed to conceptualization and performed animal surgery. H.E. contributed to conceptualization and acquired funding. T.E. contributed to conceptualization. A.P. contributed to investigation and supervision, and was involved in writing—original draft and funding acquisition. P.T. contributed to conceptualization and supervision, and was involved in writing—original draft and funding acquisition. F.A.S. contributed to conceptualization and supervision, and was involved in writing—original draft and funding acquisition.

## Supporting information



Supporting Information

## Data Availability

The data that support the findings of this study are available from the corresponding author upon reasonable request.
