# Supplementary material for: Pyrophosphate‐Containing Calcium Phosphates Negatively Impact Heterotopic Bone Quality
Source: Adv Healthc Mater. 2025 May 22;14(16):2405171. doi: 10.1002/adhm.202405171 (PMC12184083; doi:10.1002/adhm.202405171)

ADVANCED HEALTHCARE MATERIALS

Supporting Information

for *Adv. Healthcare Mater.*, DOI 10.1002/adhm.202405171

Pyrophosphate-Containing Calcium Phosphates Negatively Impact Heterotopic Bone Quality

Martina Jolic, Isabella Åberg, Omar Omar, Håkan Engqvist, Thomas Engstrand, Anders Palmquist, Peter Thomsen and Furqan A. Shah**

SUPPORTING INFORMATION

Pyrophosphate-Containing Calcium Phosphates Negatively Impact Heterotopic Bone Quality

Martina Jolic, Isabella Åberg, Omar Omar, Håkan Engqvist, Thomas Engstrand, Anders Palmquist, Peter Thomsen, Furqan A. Shah**

M. Jolic, I. Åberg, A. Palmquist, P. Thomsen, F. A. Shah
Department of Biomaterials, Institute of Clinical Sciences, Sahlgrenska Academy, University of Gothenburg, Box 412, 405 30, Gothenburg, Sweden
E-mail: peter.thomsen@biomaterials.gu.se, furqan.ali.shah@biomaterials.gu.se

O. Omar, P. Thomsen
Department of Biomedical Dental Sciences, College of Dentistry, Imam Abdulrahman Bin Faisal University, P.O. Box 1982, Dammam, 314 41, Saudi Arabia

H. Engqvist
Department of Materials Science and Engineering, Uppsala University, Box 35, Uppsala, 751 03, Sweden

T. Engstrand
Department of Molecular Medicine and Surgery, Karolinska University Hospital, Stockholm, 171 76, Sweden

Table S1. *Implanted CaP material formulations.*

	Components (in %)		
Composition	Monetite	β-TCP	Ca-PP
0% Ca-PP	90.9	9.1	0
3% Ca-PP	88.18	8.81	3
6% Ca-PP	85.45	8.54	6
10% Ca-PP	81.82	8.18	10
12.5% Ca-PP	79.55	7.95	12.5

Figure S1. *Partial overviews of undecalcified, bisected sample blocks imaged with backscattered electron scanning electron microscopy. 0% Ca-PP constructs at 12 weeks. Scale bars = 1 mm.*

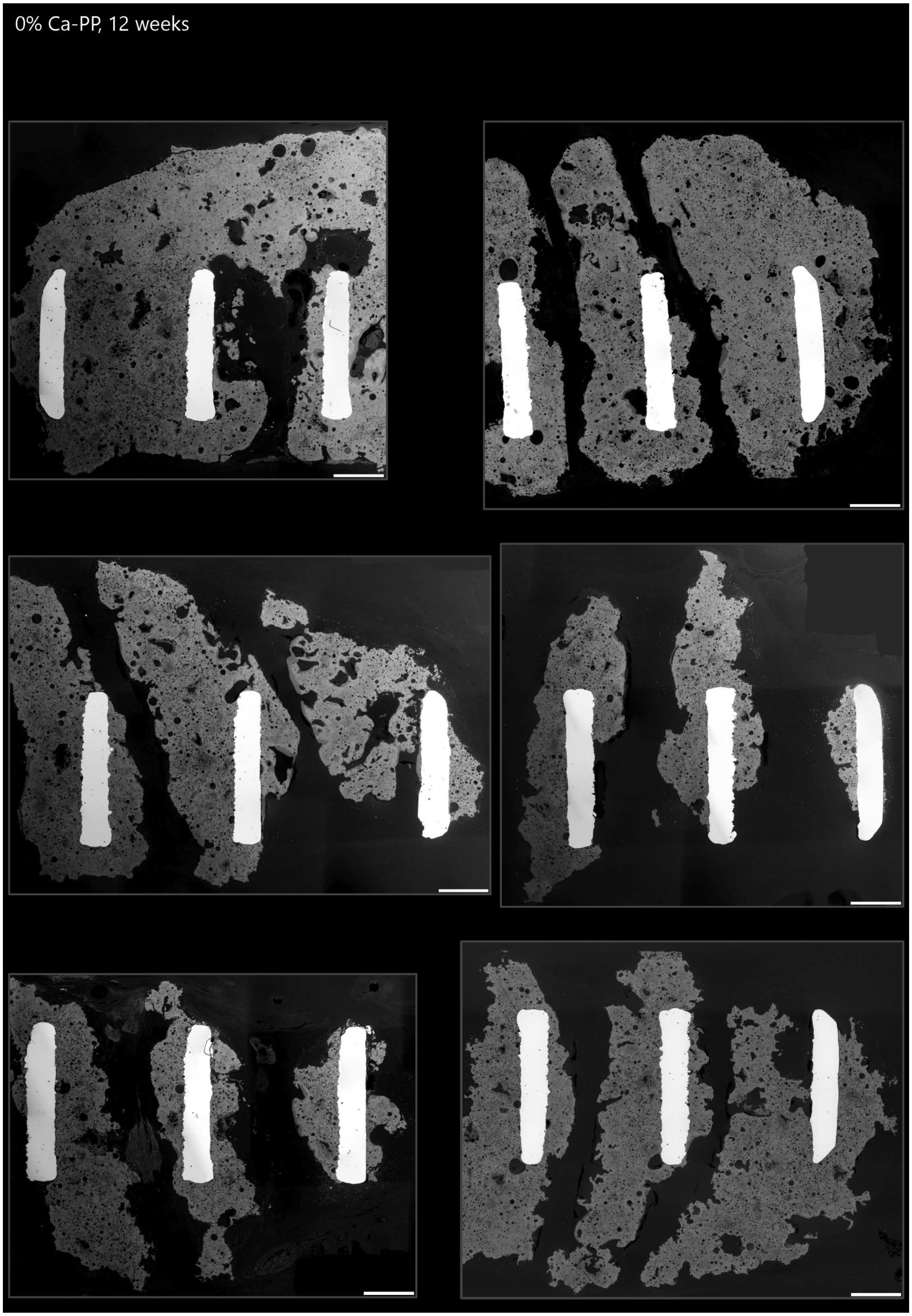

Figure S2. *Partial overviews of undecalcified, bisected sample blocks imaged with backscattered electron scanning electron microscopy. 3% Ca-PP constructs at 12 weeks. Scale bars = 1 mm.*

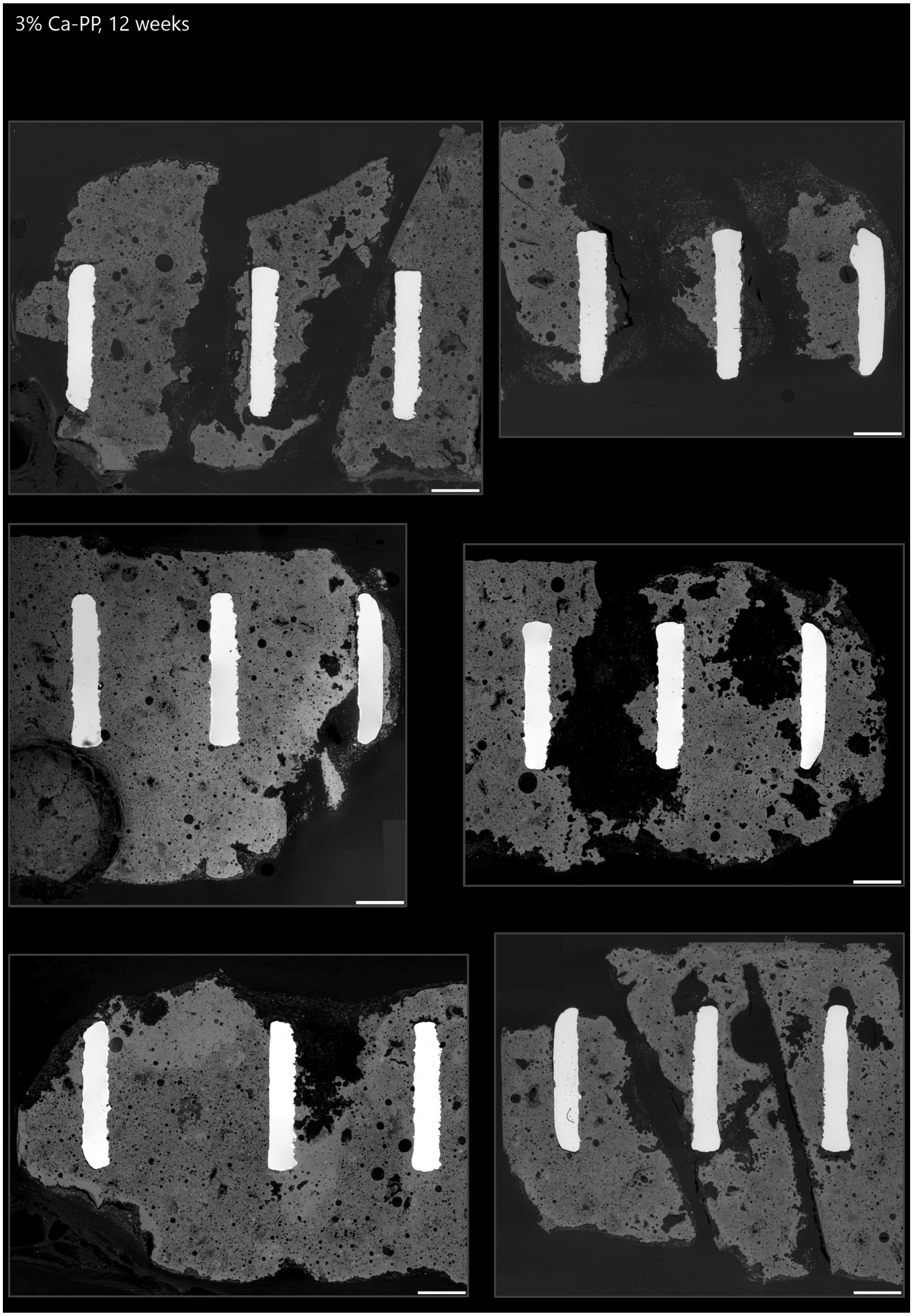

Figure S3. *Partial overviews of undecalcified, bisected sample blocks imaged with backscattered electron scanning electron microscopy. 6% Ca-PP constructs at 12 weeks. Scale bars = 1 mm.*

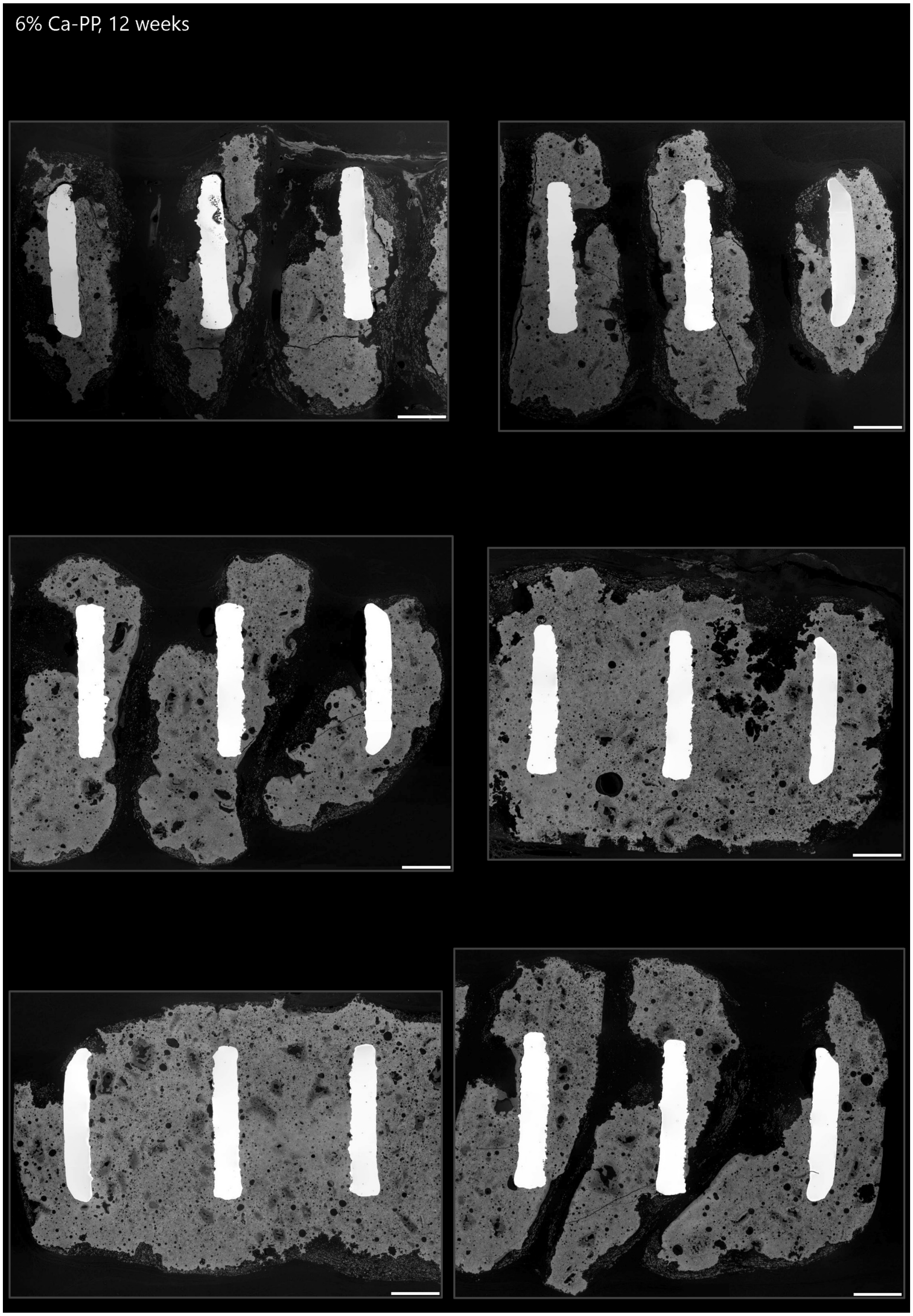

Figure S4. *Partial overviews of undecalcified, bisected sample blocks imaged with backscattered electron scanning electron microscopy. 10% Ca-PP constructs at 12 weeks. Scale bars = 1 mm.*

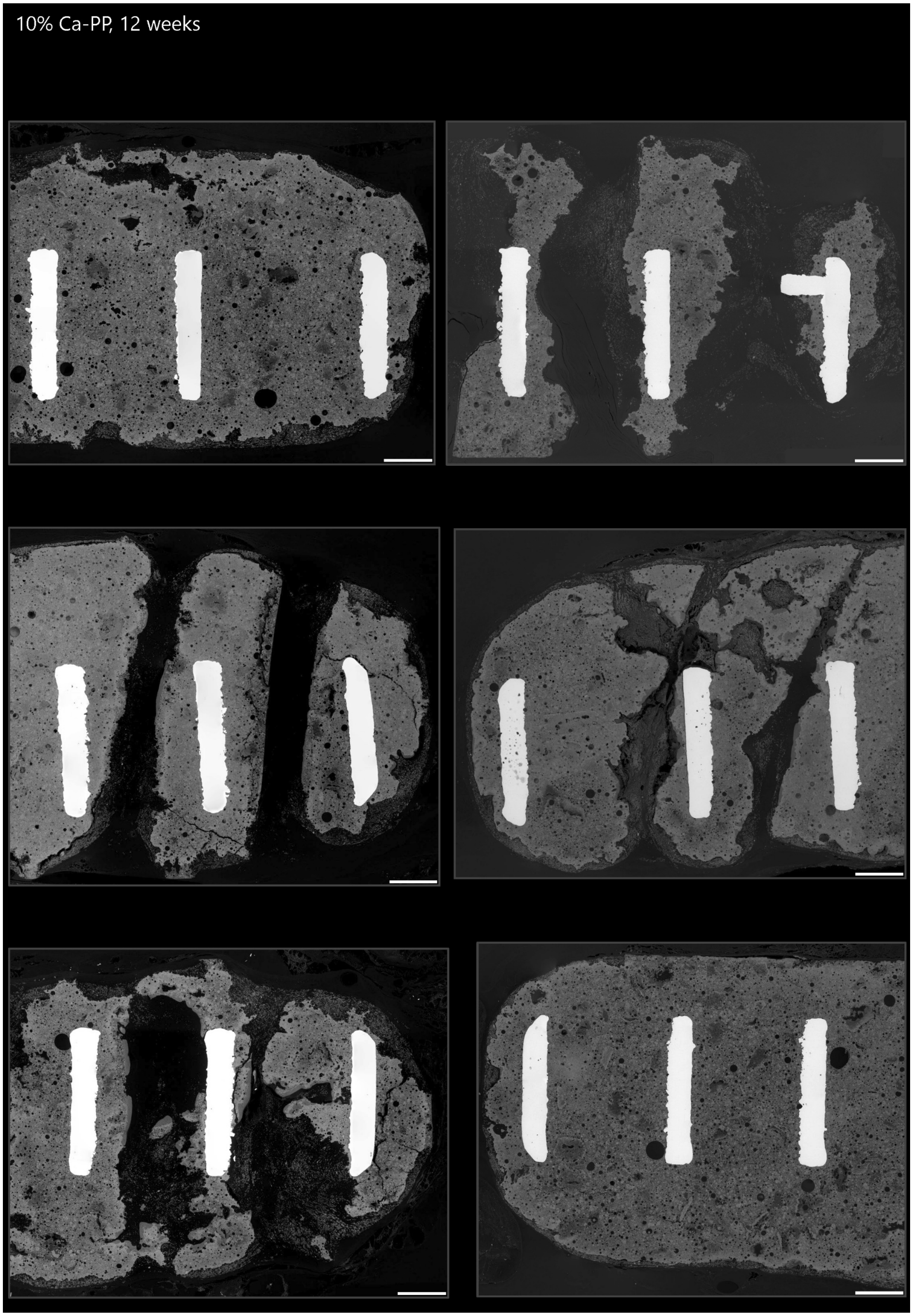

Figure S5. *Partial overviews of undecalcified, bisected sample blocks imaged with backscattered electron scanning electron microscopy. 12.5% Ca-PP constructs at 12 weeks. Scale bars = 1 mm.*

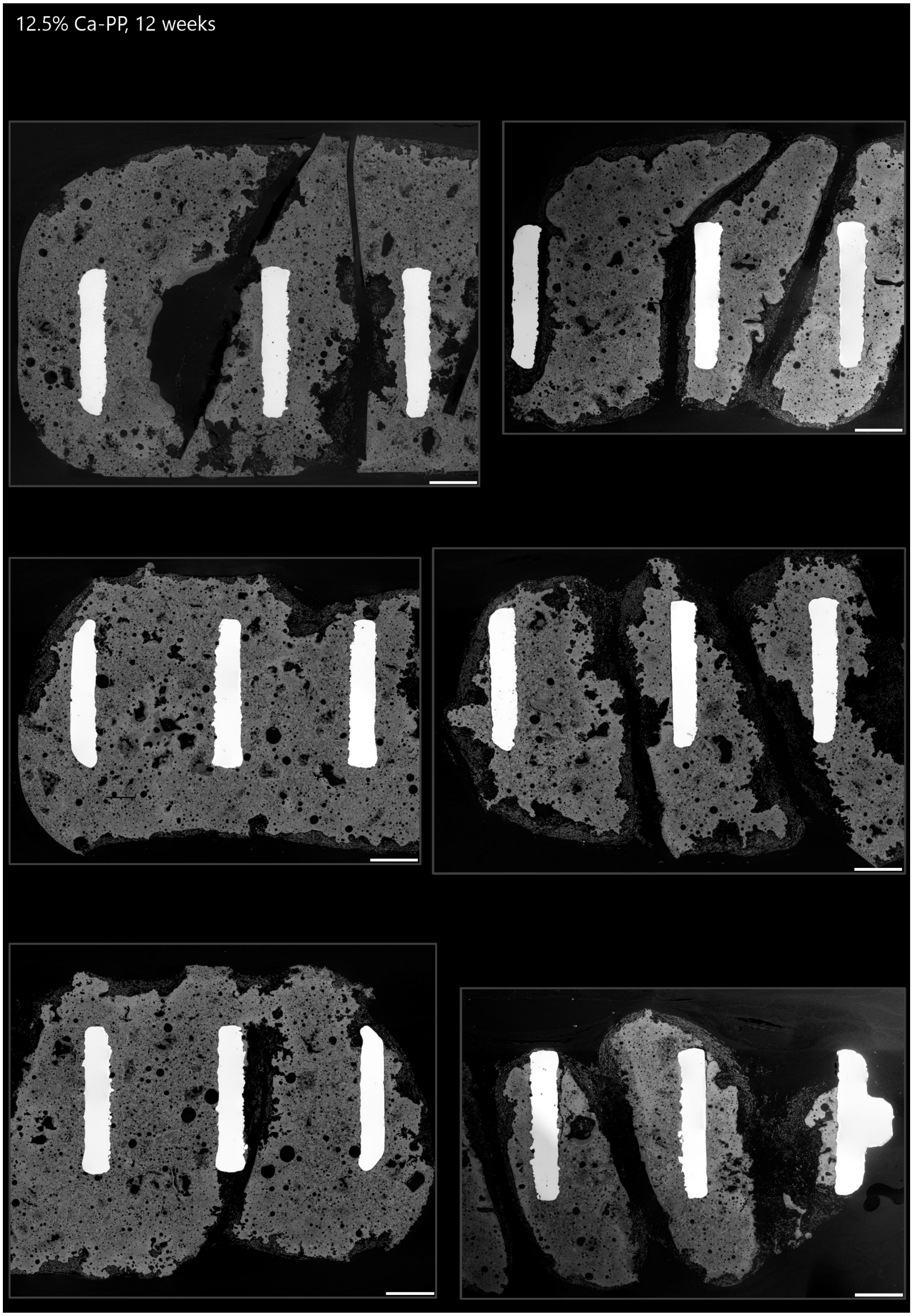

Figure S6. *Partial overviews of undecalcified, bisected sample blocks imaged with backscattered electron scanning electron microscopy. 0% Ca-PP constructs at 52 weeks. Scale bars = 1 mm.*

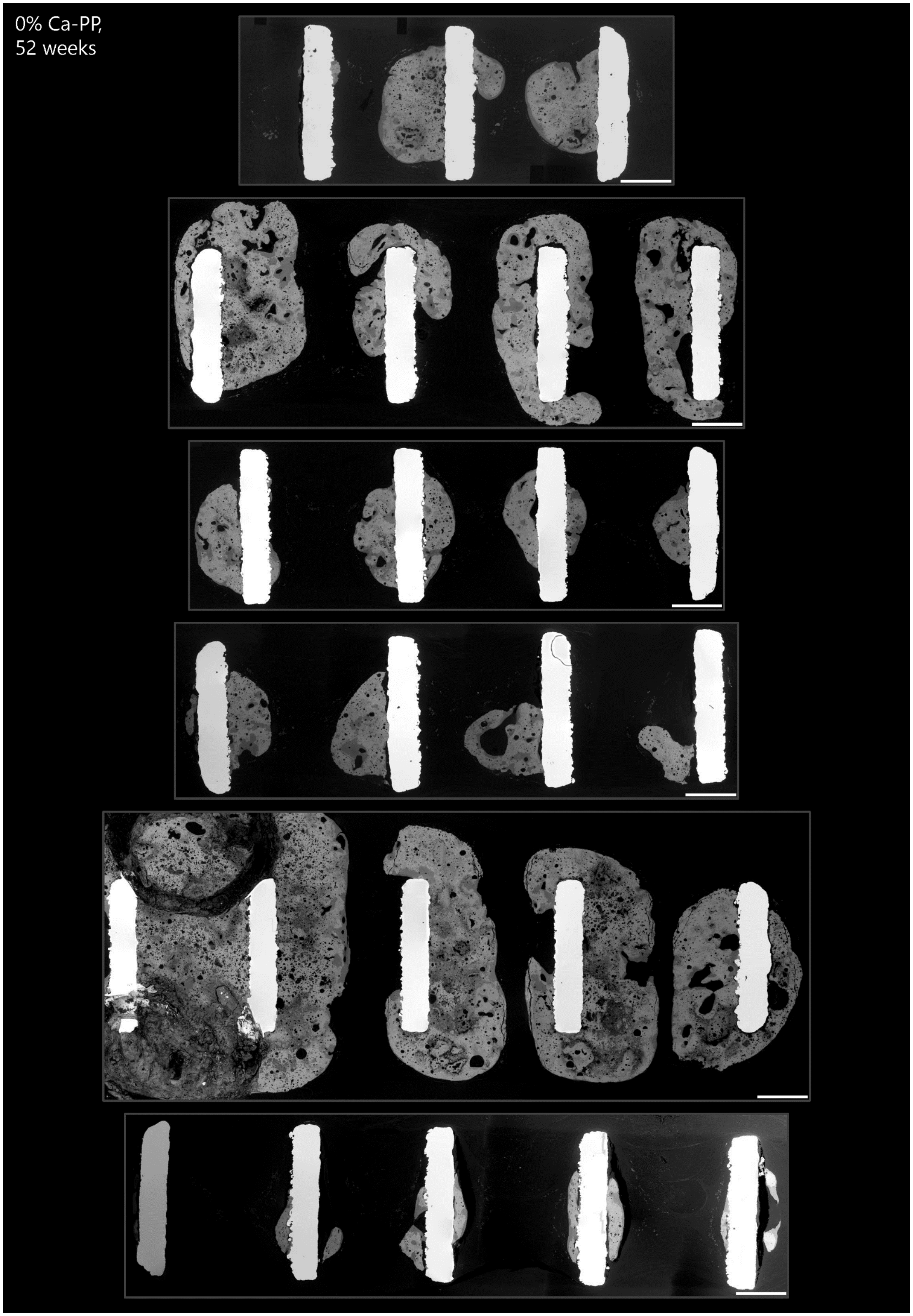

Figure S7. *Partial overviews of undecalcified, bisected sample blocks imaged with backscattered electron scanning electron microscopy. 3% Ca-PP constructs at 52 weeks. Scale bars = 1 mm.*

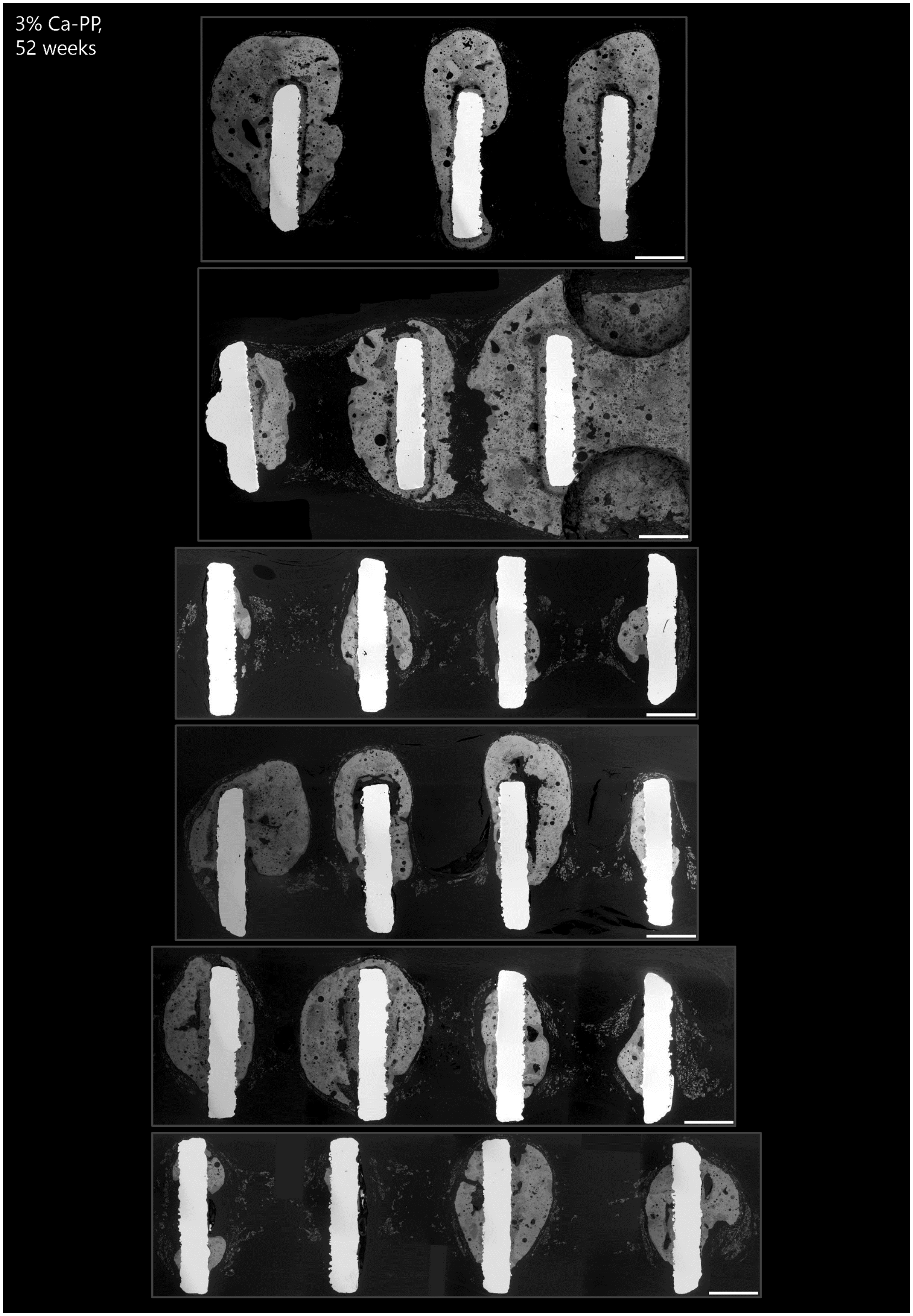

Figure S8. *Partial overviews of undecalcified, bisected sample blocks imaged with backscattered electron scanning electron microscopy. 6% Ca-PP constructs at 52 weeks. Scale bars = 1 mm.*

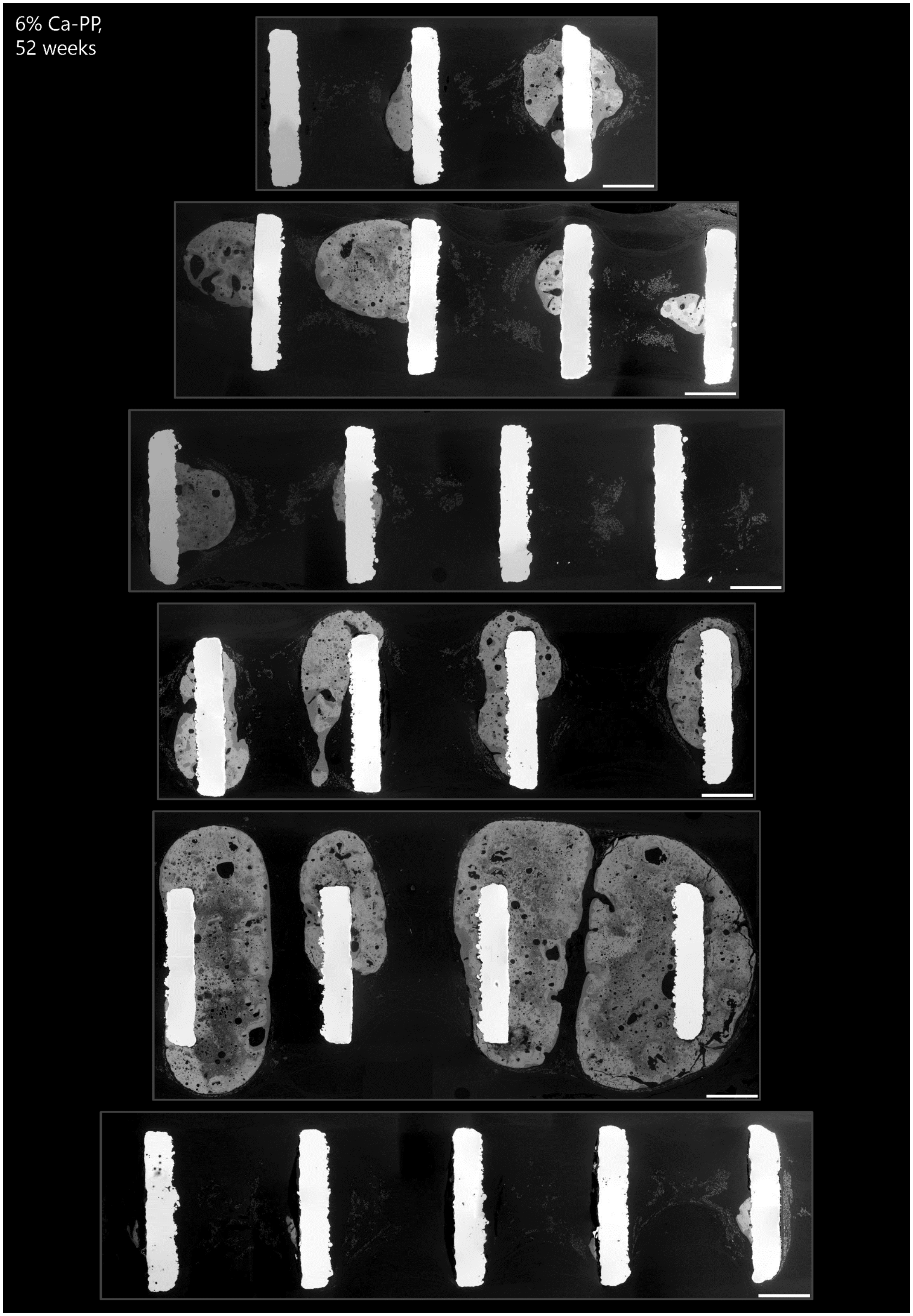

Figure S9. *Partial overviews of undecalcified, bisected sample blocks imaged with backscattered electron scanning electron microscopy. 10% Ca-PP constructs at 52 weeks. Scale bars = 1 mm.*

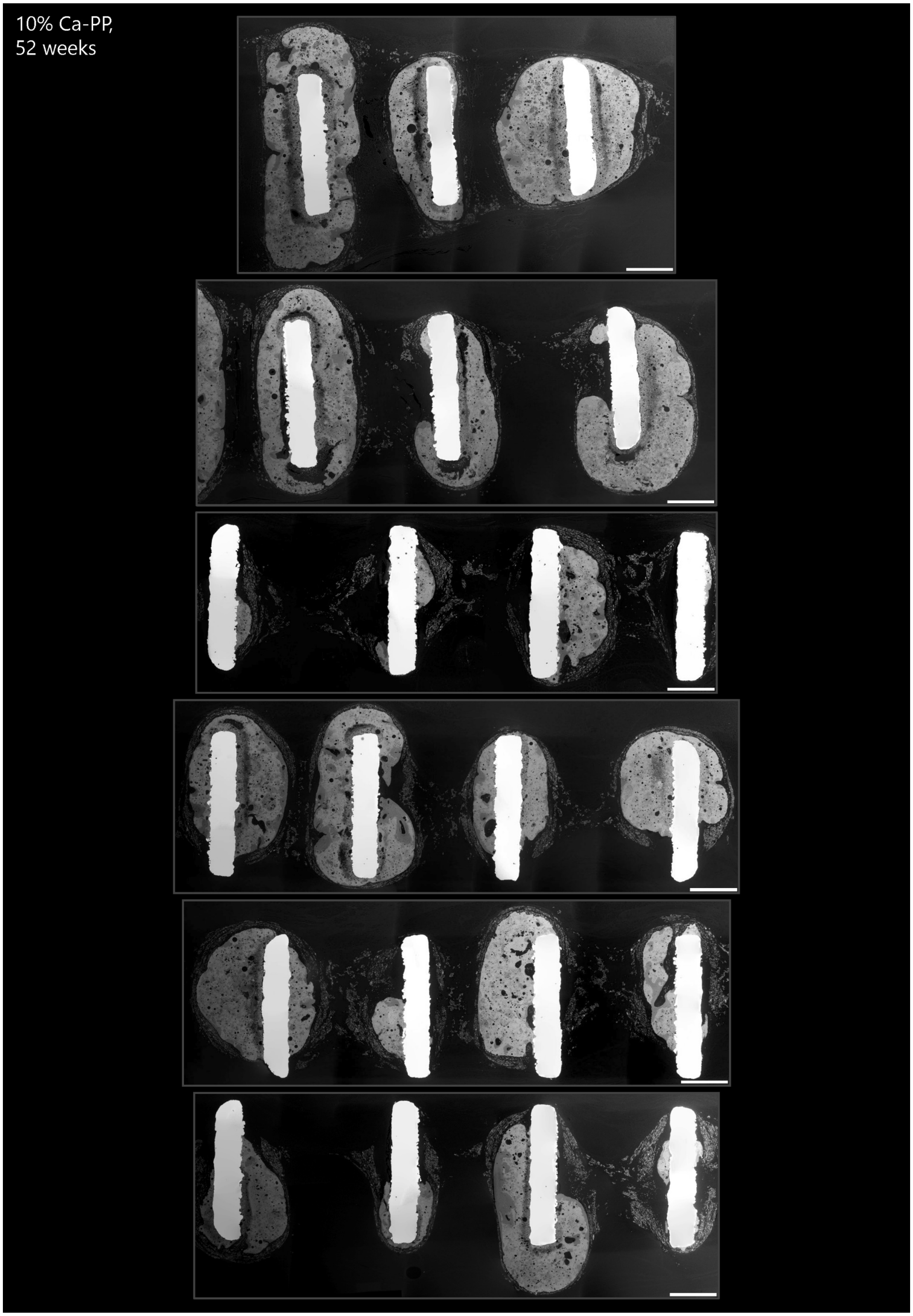

Figure S10. *Partial overviews of undecalcified, bisected sample blocks imaged with backscattered electron scanning electron microscopy. 12.5% Ca-PP constructs at 52 weeks. Scale bars = 1 mm.*

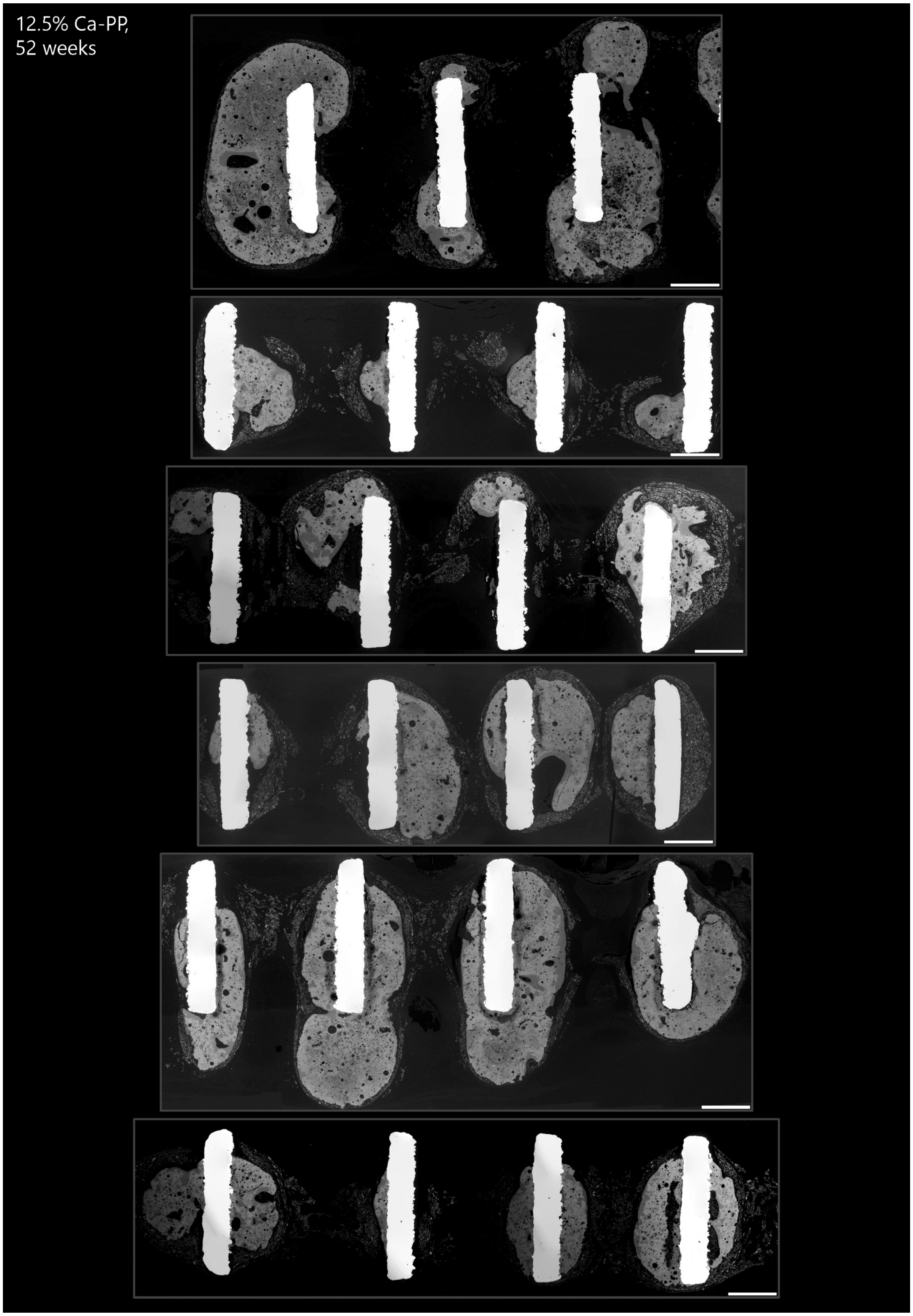

Figure S11. *Representative Raman spectra obtained from the three regions of interest: heterotopic bone, CaP at the bone interface, and CaP furthest away from the bone interface at 12- and 52 weeks in vivo. The acquired spectra were processed by performing background subtraction at 20% coarseness and cosmic ray removal with the "remove spikes" function at maximum width of 5 pixels in Spectragryph V1.2.16.1. Raw spectrum (green), background fluorescence profile (red), and corrected spectrum (blue).*

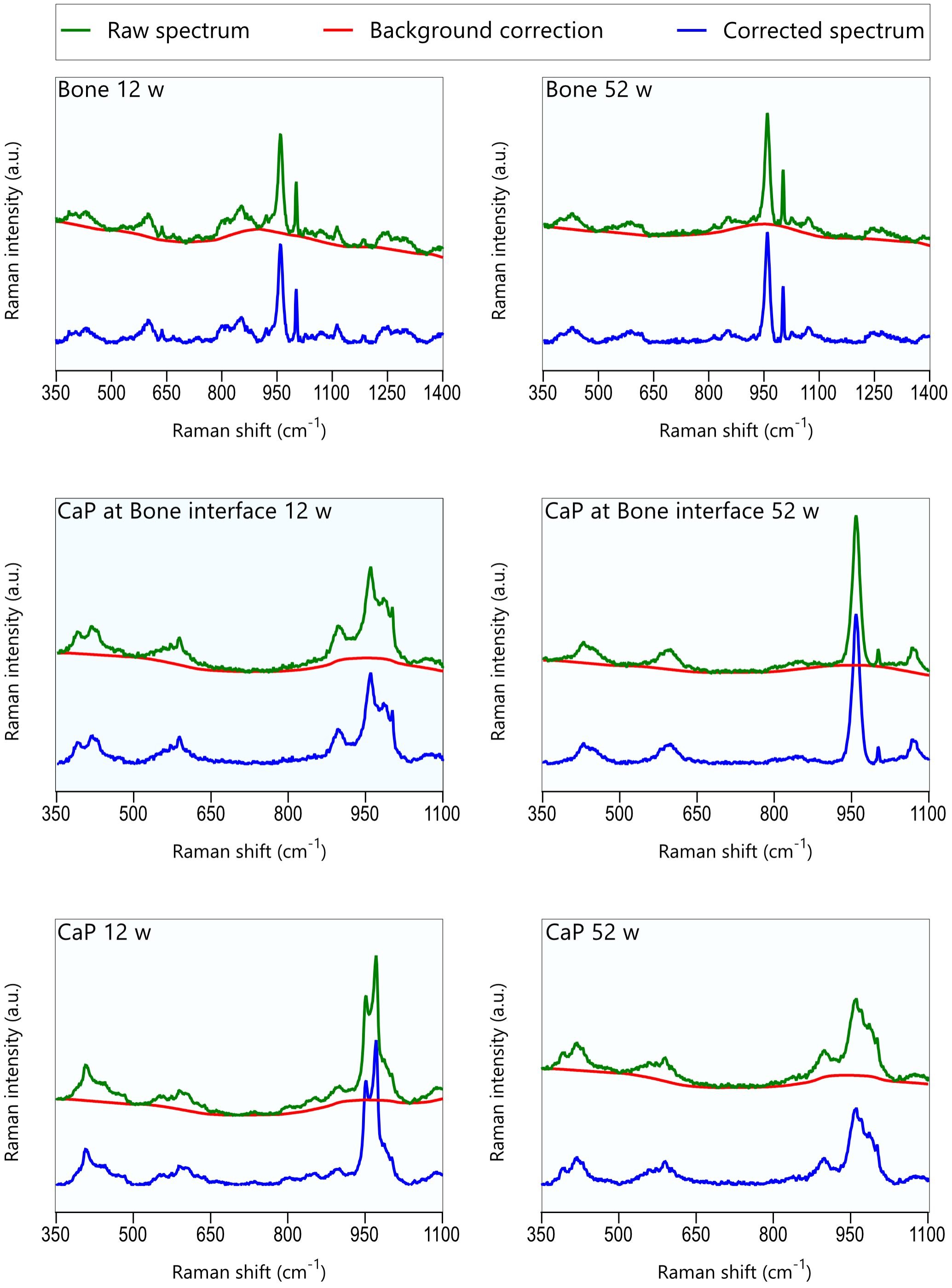

Figure S12. *Overviews of undecalcified, histological sections of control Ti6Al4V ELI implants stained with Van Gieson's stain at 12 weeks (n = 6). Scale bars = 1 mm.*

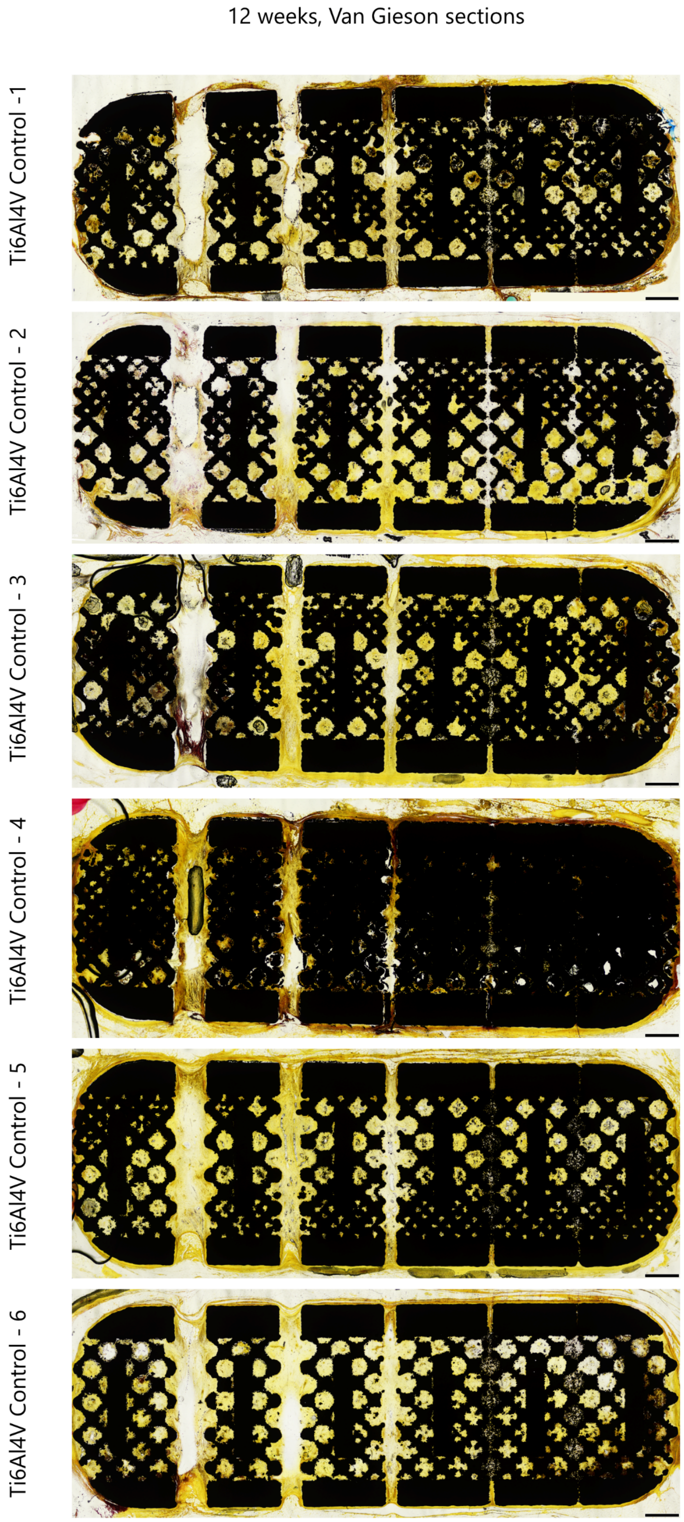

Figure S13. *Overviews of undecalcified, histological sections of control Ti6Al4V ELI implants stained with Van Gieson's stain at 52 weeks (n = 6). Scale bars = 1 mm.*

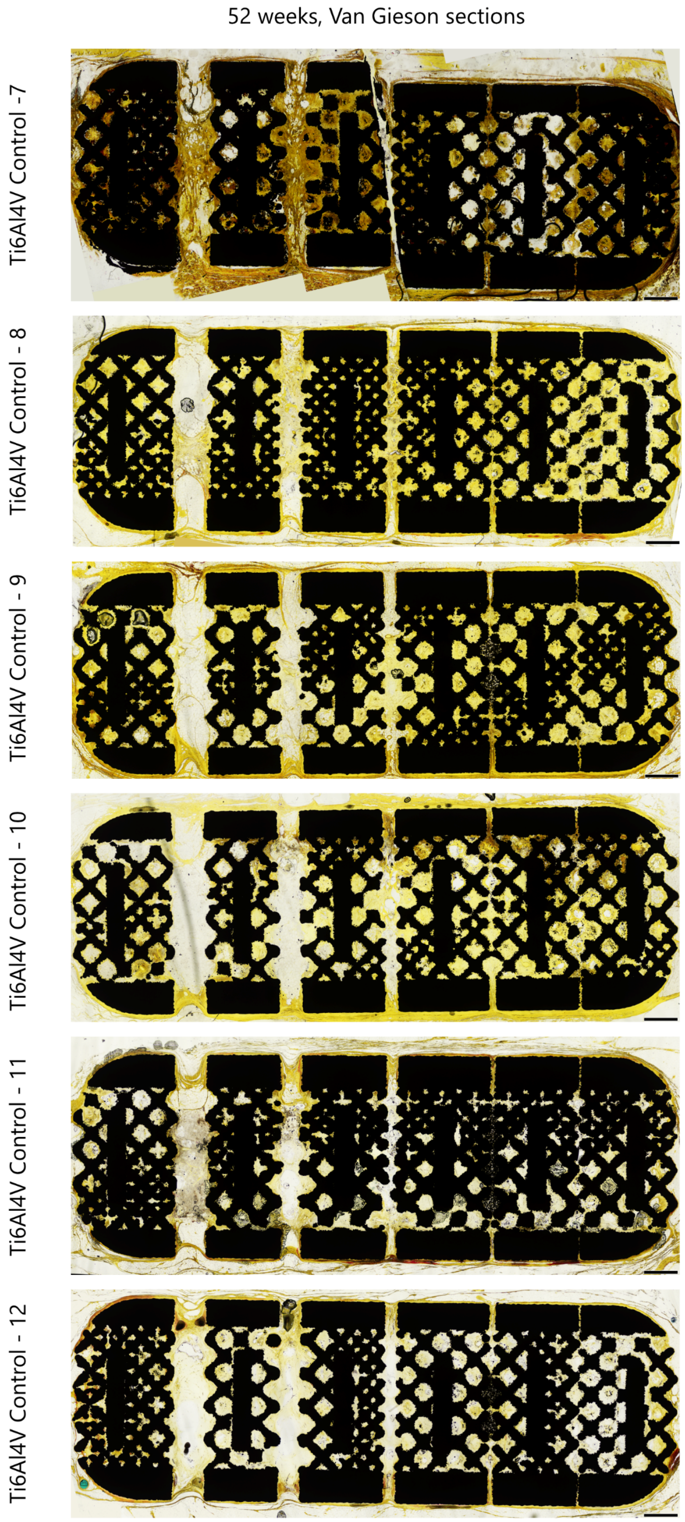

Table S2. *Measured bone areas (B.Ar).*

B.Ar (mm^2)	*12 weeks in vivo*				
Animal #	0% Ca-PP	3% Ca-PP	6% Ca-PP	10% Ca-PP	12.5% Ca-PP
1	1.12	0.23	0.78	2.43	0.51
2	0.40	0.15	0.32	0.05	0.19
3	0.38	0.07	0.18	0.29	0.45
4	0.37	1.55	0.99	0.99	0.58
5	2.64	1.10	1.29	2.96	2.04
6	0.91	0.35	0.78	1.13	0.67
	52 weeks in vivo				
7	1.43	0.55	1.01	1.73	0.55
8	0.44	3.80	0.22	1.65	1.26
9	3.04	2.09	0.79	2.77	2.19
10	3.18	2.45	2.45	2.75	5.37
11	1.15	0.75	0.39	1.40	1.21
12	0.74	1.24	0.12	1.17	0.95

Table S3. *Measured calcium phosphate areas (CaP.Ar).*

CaP.Ar (mm^2)	*12 weeks in vivo*				
Animal #	0% Ca-PP	3% Ca-PP	6% Ca-PP	10% Ca-PP	12.5% Ca-PP
1	61.83	72.77	50.06	50.21	63.21
2	36.60	24.83	35.20	45.33	49.26
3	38.72	48.59	16.02	51.32	59.13
4	48.01	59.03	36.95	53.68	46.26
5	54.32	70.42	47.38	53.62	49.15
6	26.18	27.46	38.89	51.97	46.18
	52 weeks in vivo				
7	6.72	1.66	5.13	11.00	2.93
8	4.21	35.79	1.60	10.01	7.89
9	17.55	25.79	7.50	27.51	20.67
10	35.12	24.35	41.62	26.01	25.13
11	5.37	3.23	3.05	7.92	4.83
12	1.56	7.36	0.54	3.03	7.39

Figure S14. *Energy dispersive X-ray spectroscopy (EDX) maps of representative regions of interest of phagocytized CaP debris at 12- and 52 weeks in vivo. Elemental maps for Ca, C, P confirm that the phagocytized debris is CaP. Insets: Averaged spectra of the EDX maps. Scale bars = 100 µm.*

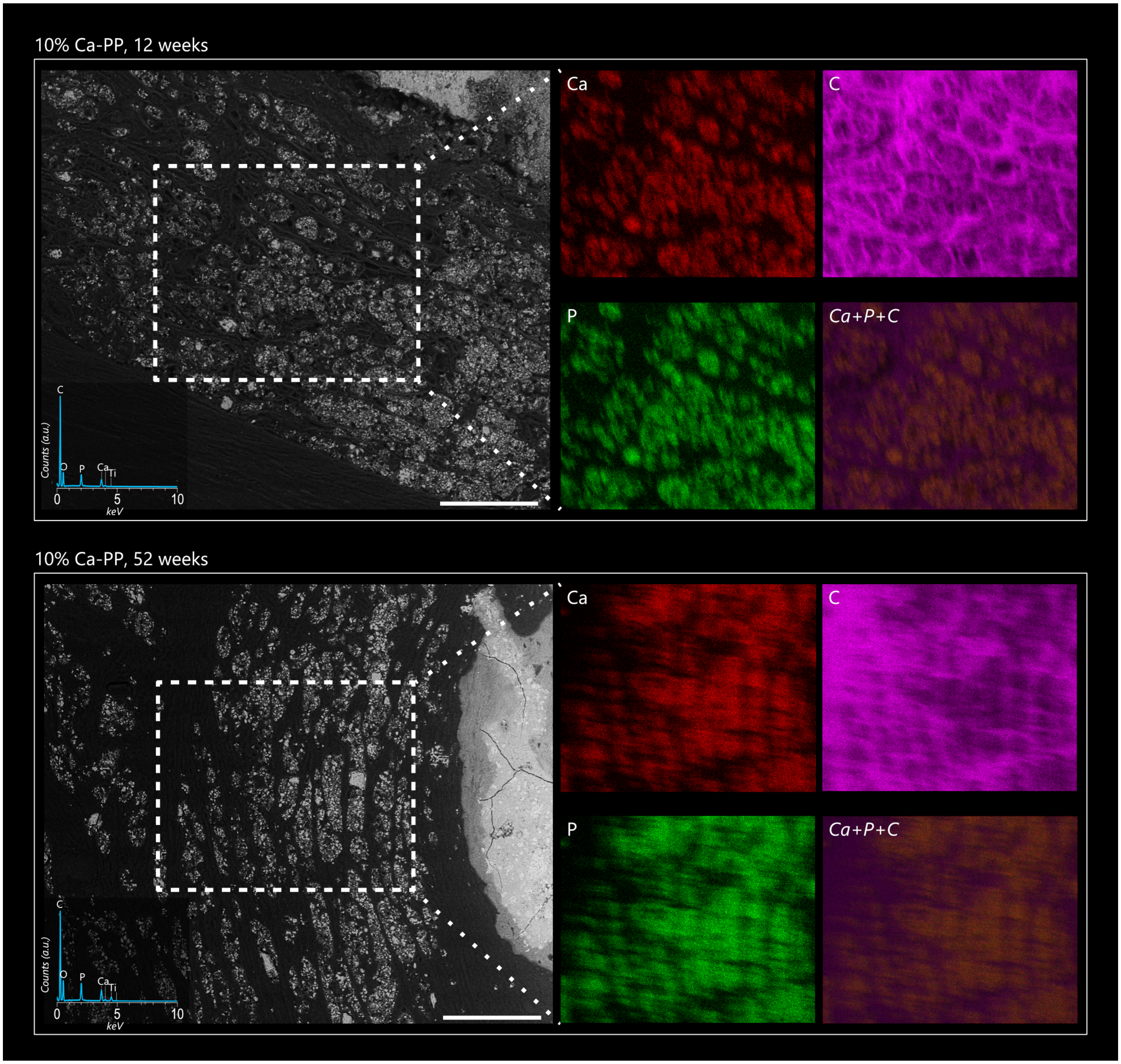

Supplement: Supplementary file 1 — Supporting Information [file ADHM-14-0-s001.pdf]
